# Self-Assembly Interactions in Magnetite-Coated Cellulose Nanocrystals: Implications for Magnetic Hyperthermia Applications

**DOI:** 10.1021/acsanm.5c05783

**Published:** 2026-04-07

**Authors:** Mohammad Jahid Hasan, Erin L. McNeill, Kishore Chand, Juganta K. Roy, Monishita Deb, Katherine Schlaak, Sydney Herzog, Yvonne Sun, Sarah Watzman, Esteban E. Ureña-Benavides, Erick S. Vasquez-Guardado

**Affiliations:** † Department of Biomedical Engineering and Chemical Engineering, 12346 The University of Texas at San Antonio, 1 UTSA Circle, San Antonio, Texas 78249, United States; ‡ Department of Chemical and Materials Engineering, University of Dayton, 300 College Park, Dayton, Ohio 45469-0256, United States; § Department of Chemistry and Physics, 14741 West Texas A&M University, Canyon, Texas 79016, United States; ∥ Department of Mechanical and Materials Engineering, 2514 University of Cincinnati, 2901Woodside Drive, Cincinnati, Ohio 45221, United States; ⊥ Department of Biology, University of Dayton, Dayton, Ohio 45469, United States; # Hanley Sustainability Institute, University of Dayton, 300 College Park, Dayton, Ohio 45469, United States

**Keywords:** Magnetic cellulose nanocrystals, TEMPO-oxidized cellulose nanocrystals, Iron oxide nanoparticles, Cellulose nanocrystals−magnetite interactions, Magnetic hyperthermia and SAR

## Abstract

Magnetic cellulose nanocrystal (MCNC) nanocomposites are promising sustainable and biocompatible platforms for magnetic hyperthermia; however, the molecular mechanisms governing Fe_3_O_4_ adsorption and deposition onto CNCs remain poorly understood. Here, sulfated (S-CNC) and TEMPO-oxidized CNCs (T-CNC) were used to prepare nanocomposites at 1:2 and 1:4 CNC:Fe_3_O_4_ mass ratios, enabling a systematic evaluation of how surface chemistry and nanoparticle loading dictate interfacial interactions and magneto-colloidal behavior. Bare magnetite nanoparticles were 21 ± 5 nm by TEM but grew to 144 ± 18 in the DLS measurement at pH 7. The S-CNC nanocomposites had hydrodynamic sizes between 144 and 210 nm, not much larger than the 140 nm long CNC rods, suggesting an enhanced dispersion stability compared to Fe_3_O_4_ alone. X-ray photoelectron spectroscopy combined with density functional theory revealed that −OH and −COOH groups drive electrostatic adsorption with charge transfer from Fe_3_O_4_ to the CNC surface, while T-CNCs showed more favorable adsorption energies and evidence of covalent Fe–O bonding. Vibrating sample magnetometry demonstrated superparamagnetic behavior for all samples, with S-CNC/Fe_3_O_4_ 1:4 and 1:2 displaying saturation magnetizations of 78 and 77 emu/g-Fe_3_O_4_, close to the 83 emu/g of bare magnetite. The T-CNC composites showed lower (60 and 66 emu/g-Fe_3_O_4_) saturation magnetizations. Zero-field-cooled/field-cooled measurements resulted in a blocking temperature of 112 K for all samples, except T-CNC/Fe_3_O_4_ 1:2 (100 K). Magnetic hyperthermia studies revealed that specific absorption rate (SAR) increased with field strength and Fe_3_O_4_ content; however, S-CNC/Fe_3_O_4_ (1:2) achieved the highest intrinsic SAR per gram of Fe_3_O_4_ (649 W/g-Fe_3_O_4_) likely due to its anisotropy and fast magnetic relaxation. Cytotoxicity assays confirmed that all nanocomposites were nontoxic toward mammalian cells. These results establish quantitative structure–property relationships between CNC surface chemistry, interfacial bonding mechanisms, and magnetic heating performance, providing a foundation for rational design of biocompatible magnetic nanocomposites for hyperthermia and related applications.

## Graphical abstract

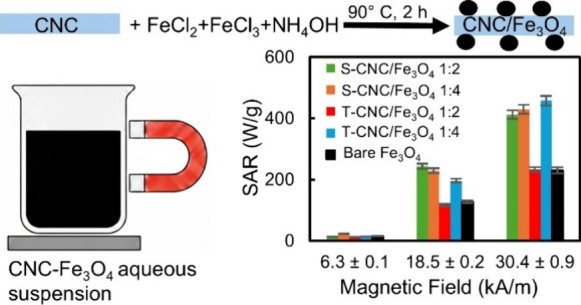

## 1 Introduction

Cellulose nanocrystals (CNCs), typically produced by controlled hydrolysis of natural cellulose, have attracted significant interest as renewable and multifunctional nanomaterials. Their unique properties, including large aspect ratio, high crystallinity, excellent mechanical strength, and a surface rich in reactive hydroxyl groups, make them highly versatile for a wide range of applications, including biomedical and biological systems (e.g., drug delivery cancer treatment and tissue engineering), environmental remediation (e.g., water treatment and pollutant removal), and the fabrication of advanced functional nanocomposites.^1,2^ Conventional sulfuric acid hydrolysis yields sulfated CNCs (S-CNCs) bearing negatively charged sulfate ester groups on their surfaces, providing exceptionally high colloidal stability over a wide pH range (∼2 and above).^3^ Typical sulfate concentration levels on CNCs range from 1 × 10^–4^ to 2 × 10^–4^ mol g^–1^-CNC, corresponding to approximately 30% of the primary −OH groups.^3^ Thus, approximately 70% of the primary hydroxyl groups remain available for further chemical modification.

A variety of strategies exist to functionalize CNC surfaces—including oxidation, acetylation, esterification, and amidation—with 2,2,6,6-Tetramethylpiperidine-1-oxyl (TEMPO)-mediated oxidation being one of the most widely adopted for the dispersion of CNCs in polar media.^4,5^ This reaction selectively converts the primary C6 hydroxyl groups of the anhydroglucose units into carboxylic acid groups under mild, aqueous conditions.^6,7^ The resulting TEMPO-oxidized CNCs (T-CNCs) retain their characteristic rod-like morphology while exhibiting substantially altered surface chemistry relative to S-CNCs. The introduction of carboxyl (−COOH) groups enhances electrostatic stabilization and broadens their suitability for subsequent functionalization and nanocomposite fabrication.

Superparamagnetic nanoparticles, especially iron oxide-based nanoparticles (i.e., SPIONs), have become important in biomedical technologies due to their magnetic responsiveness, biocompatibility, and ability to generate heat under alternating magnetic fields.^8^ Moreover, iron-based magnetic nanoparticles can be precisely manipulated, directed, and activated to target specific regions within complex media using external magnetic fields.^9,10^ This controlled movement enables their transport through dense biological matrices that are otherwise challenging to penetrate.^11,12^ Surface-functionalized magnetic nanoparticles further serve as potent hyperthermia agents, enabling spatially confined thermal activation for therapeutic delivery, cancer treatment, and antimicrobial interventions, including the disruption of bacterial biofilms.^13,14^ These multifunctional capabilities highlight the versatility of magnetic nanomaterials and motivate their integration into sustainable, biobased composite materials.

Combining CNCs with magnetite (Fe_3_O_4_) nanoparticles yields magnetic CNCs (MCNC), a promising class of nanocomposites that couple the lightweight, biobased characteristics of CNCs with the magnetic functionality of Fe_3_O_4_.^15,16^ These nanocomposites have attracted interest for applications such as targeted drug delivery, magnetic separation, imaging, and magnetic hyperthermia for cancer therapy.^17–19^ Despite this potential, the interfacial interactions between CNCs and magnetite remain poorly understood and are likely strongly influenced by CNC surface chemistry. S-CNCs and T-CNCs possess distinct anionic surface groups that can affect magnetite adsorption, nanoparticle distribution, and aggregation behavior, ultimately shaping the structural, magnetic, and functional properties of the resulting nanocomposites.

In this work, CNC/Fe_3_O_4_ nanocomposites were synthesized using S-CNCs and T-CNCs as templates at CNC-to-Fe_3_O_4_ mass ratios of 1:2 and 1:4 to investigate the influence of surface chemistry on magnetite attachment and distribution. Conductometric titration and FTIR spectroscopy confirmed successful surface modification of S-CNC to T-CNC and indicated potential interactions between both CNC types and Fe_3_O_4_ in the resulting CNC/Fe_3_O_4_ nanocomposites. XPS analysis further revealed changes in the C 1s, O 1s, and Fe 2p regions, providing insight into the nature of the interfacial binding. To complement the experiments, density functional theory (DFT), Bader charge analysis, and partial density of states (PDOS) calculations were performed to elucidate the binding mechanisms between CNC and Fe_3_O_4_, as well as to identify the oxygen sites on S-CNCs and T-CNCs that are most susceptible to Fe_3_O_4_ adsorption. The nanocomposites were additionally characterized by TEM for morphology, XRD for crystallinity, TGA for magnetite content, and VSM for magnetic behavior. Magnetic hyperthermia experiments and SAR measurements were conducted to assess their heating performance under various alternating magnetic fields for potential biomedical applications. Collectively, this combined experimental-computational approach provides a fundamental understanding of how CNC surface chemistry governs magnetite binding and guides the rational design of magnetic CNC nanocomposites for hyperthermia and related applications.

## 2 Materials and Methods

### 2.1 Materials

Sulfated cellulose nanocrystals (7.5% w/w aqueous suspension, CelluRods 100L) were purchased from CelluForce Inc. (Montreal, QC, Canada). TEMPO (>98% purity), sodium bromide (>97% purity), sodium hypochlorite solution (12% available chlorine), hydrochloric acid solution (1 N), ammonium hydroxide (28.0–30.0 w/w%), iron­(II) chloride tetrahydrate (>96% purity), and iron­(III) chloride hexahydrate (>97% purity) were all obtained from Thermo Fisher Scientific (Waltham, MA, USA) and used without further purification.

### 2.2 Synthesis of T-CNCs

TEMPO-mediated oxidation of cellulose nanocrystals (CNCs) was conducted using a modified protocol based on established methods.^4,6^ Briefly, 500 mL of a 1 wt % aqueous CNC suspension, prepared from the CelluForce stock CNC suspension, was transferred into a 1 L reaction vessel. This suspension was sonicated in an ice bath for 5 min using a probe sonicator (20 s ON/20 s OFF cycle, amplitude 30%; Q700 Sonicator, Qsonica LLC, Newtown, CT) to ensure uniform dispersion.

Next, the suspension was stirred at ambient temperature with a magnetic stirrer set to 500 rpm, while continuously monitoring the pH using a calibrated pH meter (VWR B30PCI Benchtop pH Meter, VWR, West Chester, PA). TEMPO (0.145 g) and sodium bromide (1.595 g) were added to the CNC suspension under constant stirring. The oxidation reaction was initiated by the dropwise addition of sodium hypochlorite solution (5.465 g, ∼12% available chlorine), which was preadjusted to a pH 10 using 0.1 M HCl. The pH of the reaction mixture was maintained at 10 throughout the reaction by adding 0.2 M NaOH as needed over a 4-h period.

Once the reaction was complete, it was quenched by adding 25 mL of ethanol, followed by an additional 30 min of stirring. The pH of the suspension was then adjusted to 7 using 0.1 M HCl. The oxidized CNC suspension was dialyzed against deionized water for 3 days using dialysis tubing (MWCO: 12–14 kDa), with daily water changes to remove residual reagents. After dialysis, the purified T-CNCs were collected, transferred to an airtight storage bottle, and stored at 4 °C for further use.

### 2.3 Synthesis of Magnetic Cellulose Nanocomposites

Magnetic cellulose nanocrystals (CNC/Fe_3_O_4_) were synthesized via a coprecipitation method using iron chloride salts and ammonium hydroxide (NH_4_OH) in the presence of either sulfated or TEMPO-oxidized CNCs at CNC-to-magnetite mass ratios of 1:2 or 1:4.^20^ For the 1:4 formulation, a 400 mL aqueous CNC suspension (0.469 wt %) was prepared from the 3.10 wt % CNC stock suspension and probe-sonicated for 5 min (amplitude: 30%, 1 min on/1 min off cycle) in an ice bath, followed by degassing with nitrogen gas for 5 min. Separately, 6.4 g of iron­(II) chloride tetrahydrate and 15.78 g of iron­(III) chloride hexahydrate were dissolved in 100 mL of deionized (DI) water and similarly degassed under nitrogen. The iron salt solution was added dropwise to the CNC suspension under a continuous flow of nitrogen in a round-bottom flask fitted with a condenser. The mixture was stirred using an overhead stirrer at 1000 rpm and maintained at 90 °C for 2 h. Following this, 75 mL of NH_4_OH was added dropwise, resulting in an immediate color change to black, indicating the formation of magnetite. The reaction was allowed to proceed for an additional 2 h under the same conditions. After the reaction, the mixture was cooled to room temperature, transferred to a beaker, covered with parafilm, and placed in a biosafety cabinet to maintain sterile conditions. Magnetic separation was conducted inside the biosafety cabinet using a 2″ × 2″ × 1″ neodymium permanent magnet (Grade N52, 14,800 G, Applied Magnets, Plano, TX, USA), and the supernatant was carefully decanted. The magnetic product was washed 5–6 times with sterile DI water until the supernatant pH approached neutrality (pH ∼7). The purified nanoparticles were redispersed in sterile degassed DI water, purged with nitrogen gas for 2 min using a flame-sterilized syringe, sealed in an airtight container, and stored in a vacuum cabinet for future use. For the 1:2 CNC/Fe_3_O_4_ formulation, a 400 mL CNC suspension (0.937 wt %) was prepared by diluting 120.903 g of the 3.10 wt % CNC stock suspension with 279.097 g of DI water. All subsequent steps were identical with the 1:4 ratio synthesis protocol.

### 2.4 Characterization of the Nanoparticles

Transmission electron microscopy (TEM) imaging of the nanoparticles was performed by drop-casting dilute nanoparticle suspensions (0.001 wt %) onto carbon-coated copper grids (CF300-Cu, Electron Microscopy Sciences, Hatfield, PA, USA), followed by overnight drying at room temperature. Imaging was carried out using a Hitachi S-5500 Field Emission Scanning Electron Microscope (Hitachi Ltd., Tokyo, Japan) operated in both SEM mode and BF-STEM mode. Energy-dispersive X-ray spectroscopy (EDS) mapping was performed on the corresponding images to identify the elemental composition. The presence and spatial distribution of iron (Fe) and oxygen (O) were analyzed to confirm the localization of Fe_3_O_4_ nanoparticles on the CNC rods.

The thermal decomposition behavior of S-CNC, T-CNC, bare Fe_3_O_4_ nanoparticles, and CNC/Fe_3_O_4_ nanocomposites was investigated using a thermogravimetric analyzer (TGA) (Q500, TA Instruments, New Castle, DE, USA). About 5–10 mg of dried samples were heated from room temperature to 800 °C at a heating rate of 10 °C per minute under a nitrogen atmosphere. From this analysis, the residual weight (RW) at 600 °C provided an estimation of the Fe_3_O_4_ amount in the S-CNC/Fe_3_O_4_ and T-CNC/Fe_3_O_4_ composites based on the procedure described in our previous publication.^20^ A detailed procedure for the calculation of magnetite content in the magnetic CNCs is provided in the Supporting Text S1 and Table S1.

Powder X-ray diffraction (XRD) analysis was performed using a Malvern Panalytical Empyrean Nano Edition diffractometer (Malvern Panalytical, Malvern, UK). Nanoparticle dispersions were freeze-dried prior to analysis, and diffraction patterns were recorded over a 2θ range of 10° to 80° using Cu Kα radiation (λ = 0.1540 nm). Data were collected at room temperature (25 °C) with a step size of 0.003° and a step time of 0.6 s per step, while the sample was rotated at 4 rpm to ensure uniform exposure.

The XRD peaks were further analyzed using the Williamson–Hall method to estimate crystallite size and lattice strain.^21^ In this approach, the full width at half-maximum (fwhm) of each diffraction peak (β) is related to crystallite size (D) and microstrain (ε) by the equation:

$$\beta \cdot \cos\theta =\frac{k\cdot \lambda }{D}+4\cdot \varepsilon \cdot \sin\theta$$

where θ is the Bragg angle, λ is the X-ray wavelength (λ = 0.1540 nm), and k is the shape factor (0.9). A plot of β cosθ versus 4 sinθ allows simultaneous determination of crystallite size from the intercept and microstrain from the slope. The intrinsic broadening (β) was determined by subtracting the instrumental broadening from the observed broadening using the relation $\beta =\sqrt{\left(\beta_{obs}^{2}-\beta_{inst}^{2}\right)}$ .

Attenuated total reflectance Fourier-transform infrared (ATR-FTIR) spectroscopy was performed on freeze-dried nanoparticle samples using a Shimadzu IRAffinity-1S spectrometer (Shimadzu Corporation, Kyoto, Japan). Spectra were recorded with a total of 100 scans to ensure adequate signal-to-noise ratio.

Dynamic light scattering (DLS) and zeta potential measurements were carried out using a Malvern Zetasizer Nano ZS instrument (Malvern Panalytical, Malvern, UK). Nanoparticle suspensions were prepared at concentrations of 0.01 wt % for DLS and 0.1 wt % for zeta potential analysis, with all measurements conducted over a pH range of 3 to 9.

Vibrating sample magnetometry (VSM) measurements were performed on powder samples using a Quantum Design Physical Property Measurement System (PPMS) DynaCool (Quantum Design, Inc., San Diego, CA, USA). The powder samples were kept inside of a polypropylene container; then the assembly was placed in a brass canister, which was inserted inside the PPMS chamber for the measurements. Magnetization data were collected at room temperature (∼23 °C) over an applied magnetic field range of −30,000 to +30,000 Oe. The magnetic field ramp rate was 100 Oe/s. Measurements were conducted under near-vacuum conditions at working pressures below 5 Torr. Furthermore, zero-field-cooled (ZFC) and field-cooled (FC) magnetization measurements were performed in an applied field of 100 Oe over a temperature range of 3–390 K with cooling and heating rates of 5 K min^–1^. For ZFC measurements, the samples were first cooled from 390 to 3 K in the absence of an external magnetic field, and the magnetization was recorded during warming in the presence of the applied field. For FC measurements, the samples were cooled from 390 to 3 K in the presence of the applied field (100 Oe), and magnetization was measured during warming.

### 2.5 First-Principles Modeling

To get insight into the adsorption behavior and bonding nature of magnetite (Fe_3_O_4_) on the two types of CNCs we used plane-wave based density functional theory (DFT) approaches using Vienna *ab initio* Simulation Package (VASP 6.4).^22–24^ van der Waals density functional^25^ (vdW-DF2 version), with a more accurate semilocal exchange functional, was used under the generalized gradient approximation (GGA). The vdW-DF2 functional was able to reproduce the bulk properties of the CNC (Table S2), which were modeled as cellulose Iβ crystals, and these results are comparable to previous reported results.^26^ In all DFT calculations, PAW pseudopotentials were used to model the interaction between the valence electrons and the core, whereas plane-wave basis sets with an energy cutoff of 450 eV were used to expand the Kohn–Sham orbitals. The valence electrons of the atoms were described as Fe (3d^7^4s^1^), C (2s^2^2p^2^), O (2s^2^2p^4^) and H (1s^1^).

Based on our XRD observations, we modeled three different surfaces, namely, (110), (100) and (200) of CNC and computed the surface formation energies, E_surf_^f^ (Table S3). Due to the low symmetry of cellulose Iβ, it surfaces has distinguished properties, for instance, the (100) and (200) surfaces, and the (110) and (1–10) surfaces, are equivalent.^27,28^ Our calculation revealed that the (100) and (200) surface has similar E_surf_^f^ and surface termination. Similar results have been reported in the literature with all the reported work based on the CNC (100) surface.^27–29^ Based on our XRD, DFT and literature, we model our magnetite-CNC interface system using the CNC (100) surface which is parallel to the cellulose sheet. Pristine S-CNC (100) and T-CNC (100) are described in Supporting Information Figure S1a,b. The CNC (100) surface was modeled with a (1 × 1) periodically repeated slab consisting of five layers and 214 atoms. Additionally, a 15 Å vacuum layer between the slabs was utilized to reduce the interaction between them. The bottommost three layers were fixed to mimic the bulk properties of CNC. The Brillouin zone integration was sampled with a Γ centered (4 × 3 × 1) Monkhorst–Pack k-mesh. Self-consistent DFT energies were converged to 10^–7^ eV and all the atoms were allowed to relax until the forces were less than 0.03 eV/Å with Gaussian smearing (σ = 0.05 eV). The projected density of states (PDOS) along with the crystal orbital Hamilton population (COHP)^30^ was calculated using the software package Local Orbital Suite Toward Electronic-Structure Reconstruction (LOBSTER). The absolute charge spilling is lower than 1.46% for all the structures. VESTA^31^ was used to visualize the structure and plot charge density differences. Moreover, to explore the stability of the structure of CNC(100)/Fe_3_O_4_ models, the adsorption energy (*E*_ads_) of the cluster adsorbed on the CNC(100) surface was calculated by the following equation:

$$E_{\mathrm{ads}}=E_{S-\mathrm{CNC}/Fe_{3}O_{4}\;\mathrm{or T}-\mathrm{CNC}/Fe_{3}O_{4}}-E_{S-\mathrm{CNC or T}-\mathrm{CNC}}-E_{Fe_{3}O_{4}}$$

where *E*_S–CNC/Fe_3_O_4_ or T–CNC/Fe_3_O_4__, *E*_S–CNC or T–CNC_, and *E*_Fe_3_O_4__are the energies of the system S-CNC or T-CNC with adsorbed cluster, S-CNC or T-CNC slab, and cluster, respectively.

### 2.6 Magnetic Hyperthermia and SAR Measurement

The magnetic hyperthermia measurements of the CNC/Fe_3_O_4_ nanocomposites were conducted using an alternating magnetic field (AMF) system (Model 3–135/400–6, Life Systems, Atlantic Blvd., Auburn Hills). The concentration of all magnetic particle dispersions was maintained at 0.3 wt % to allow direct comparison. The heating performance of the nanocomposites, quantified by the specific absorption rate (SAR), was determined from the initial slope (first 30 s) of the temperature–time (*T*–*t*) curves (Δ*T*/Δ*t*) using the equation below:^32^

$$SAR=\frac{\rho \cdot C_{w}}{{Mass}_{MNP}}\left(\frac{\Delta T}{\Delta t}\right)$$

where ρ is the density of the colloid, *C*_w_ is the specific heat capacity of water (4.185 kJ·kg^–1^·K^–1^) and Mass_MNP_ is the concentration of the magnetic nanoparticles in suspension.

The experiments were carried out using an alternating magnetic field of frequency (f) 155 ± 4 kHz, with field amplitudes (H) of 6.3 ± 0.1, 18.5 ± 0.2, and 30.4 ± 0.9 kA/m. These conditions satisfy Hergt’s clinical safety criterion (H × *f* ≤ 5 × 10^9^ A·m^–1^·s^–1^), ensuring that the applied field and frequency are within the safe range for potential biomedical hyperthermia applications.^33^

The magnetic field in the hyperthermia experiment was determined based on the frequency used in the instrument (155 ± 4 kHz), the voltage in the coil and other instrumental parameters, using the equation below:

$$H\left(\frac{kA}{m}\right)=\frac{U\cdot 1000}{f\cdot 0.74\cdot 1.256}$$

where *H* is the magnetic field (kA/m), *U* is the root mean squared voltage (V), and *f* is the frequency (Hz). The factor 0.74 in the denominator is the sensitivity factor of the voltage probe, and 1000/1.256 is a unit conversion factor.

### 2.7 Toxicity (LDH and MTT) Measurement

Lactate dehydrogenase (LDH) is an intracellular enzyme of mammalian cells and the activity of LDH in the culture supernatant is therefore used as a proxy for cellular damage. RAW 264.7 macrophages (ATCC TIB-71) were seeded into 96-well culture plates at a density of 4 × 10^4^ cells per well and allowed to adhere for approximately 18 h. Following adherence, the cells were exposed to nanoparticles at a final concentration of 20, 100, or 500 μg/mL and subsequently incubated for 4 h at 37 °C in a humidified atmosphere containing 5% CO_2_. Two independent experiments, each containing 4 replicates for all control and treatment samples, were performed.

LDH activity was evaluated using the ApexBio LDH Cytotoxicity Assay Kit (Cat. No. K2228) following the manufacturer’s protocol. Briefly, 20 μL of the culture supernatant in each treated sample was transferred to a new 96-well plate and mixed with 30 μL of sterile water. This sample acquisition was performed very carefully to remove only the top 20 μL of sample to avoid disruption of the adherent cells or disturbance of the settled nanoparticles. Then, 50 μL of reaction mixture was added to each well, followed by incubation for 30 min at room temperature in the dark. LDH activity was quantified by measuring absorbance at 490 nm (product of LDH activity) and 680 nm (background absorbance) and subtracting the absorbance value measured at 680 nm from absorbance value measured at 490 nm.

To validate the LDH measurements, we also performed a standard MTT reduction assay, based on the activity of viable and active cells to reduce the tetrazolium salts [MTT, (3-(4,5-dimethylthiazol-2-yl)-2,5-diphenyltetrazolium bromide]. After 24 h of nanoparticle treatment, RAW 264.7 macrophages were washed three times with phosphate-buffered saline (PBS) supplemented with calcium and magnesium. Subsequently, 50 μL of MTT solution (0.5 mg/mL; Thermo Scientific, Cat. No: L11939.06) and 200 μL of PBS were added to each well, and the plates were incubated for 4 h at 37 °C in a humidified 5% CO_2_ incubator. The RAW 264.7 macrophages remained adherent through the washing and incubation steps. After incubation, the MTT-containing solution was discarded and replaced with 200 μL of dimethyl sulfoxide (DMSO; Fisher, BP231–1) and 25 μL of glycine buffer (0.1 M, pH 10.2) to solubilize the reduced MTT compound. After 10 min of solubilization at room temperature, the plates were centrifuged at 2,000 rpm for 5 min to precipitate all nanoparticles and cells that could interfere with the absorbance reading. The supernatant, containing the solubilized and reduced MTT, was transferred to fresh 96-well plates for absorbance measurement at 550 nm. The absorbance values of no nanoparticle control samples were used for comparison to other samples and percentages were calculated to represent cell viability.

## 3 Results and Discussion

### 3.1 Synthesis of CNC/Fe_3_O_4_ Nanocomposites

CNC/Fe_3_O_4_ nanocomposites were synthesized via a one-step coprecipitation reaction in the presence of CNCs, iron salts, and NH_4_OH, using both S-CNC and T-CNC (Scheme 1). T-CNC was first prepared from commercially obtained S-CNC (Scheme 1b). The carboxyl (−COOH) content of T-CNC, determined by conductometric titration, was 306 mmol COOH per kg of T-CNC, which is consistent with reported literature values.^4^ Detailed procedures and the titration curve are provided in Supporting Information (Text S2 and Figure S2).

**Scheme 1:**
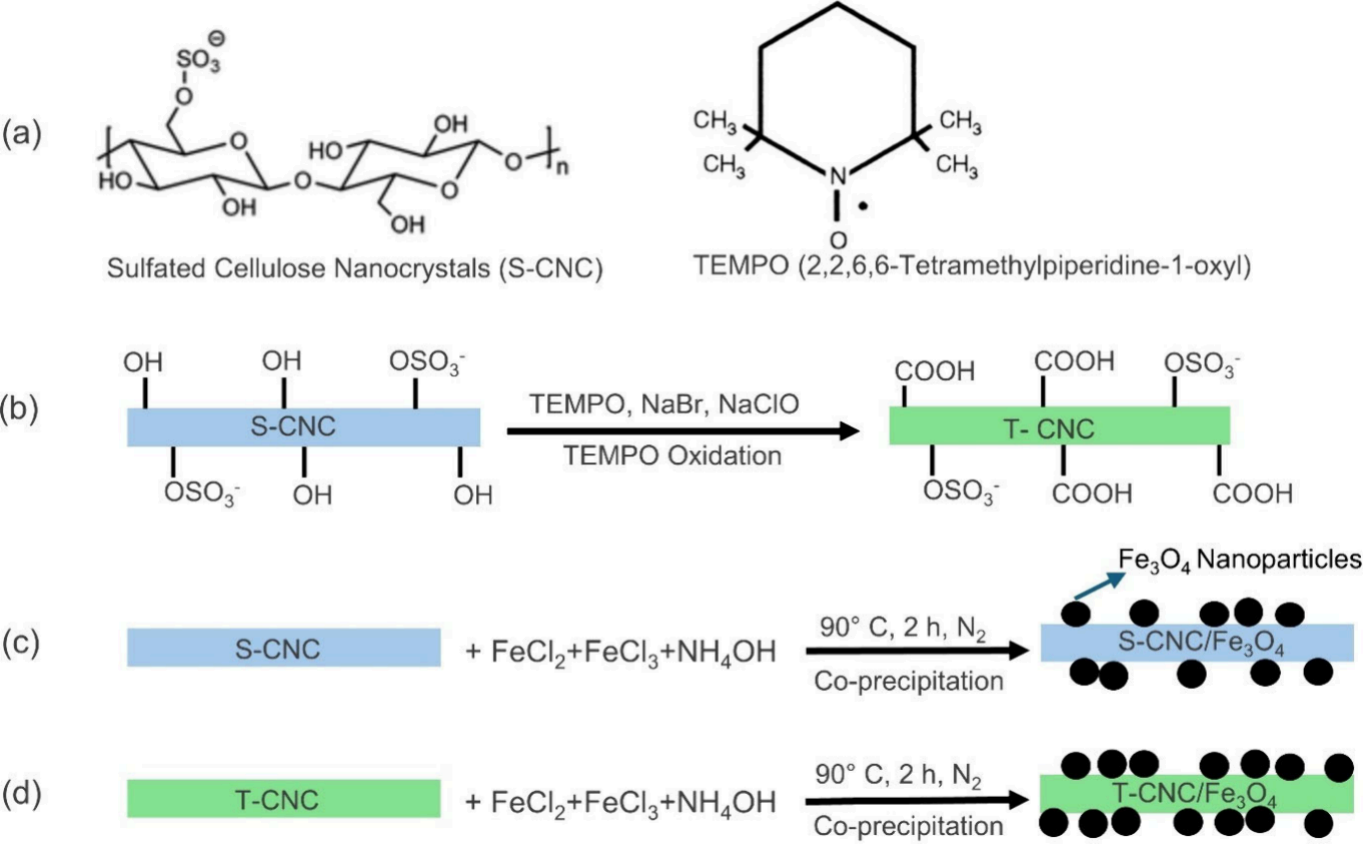
(a) Chemical Structure of S-CNC and TEMPO; (b) Surface Modification of S-CNC to T-CNC; (c) Synthesis of S-CNC/Fe_3_O_4_ Nanocomposites; (d) Synthesis of T-CNC/Fe_3_O_4_ Nanocomposites_._

As a preliminary approach, CNC/Fe_3_O_4_ nanocomposites were prepared by physically mixing presynthesized CNC dispersions (S-CNC and T-CNC) with bare Fe_3_O_4_ nanoparticles at CNC:Fe_3_O_4_ mass ratios of 1:2 and 1:4, followed by magnetic washing. Transmission electron microscopy observations (Supporting Information, Figure S3) showed no detectable CNCs remaining after magnetic separation, indicating that the CNCs were not stably associated with the Fe_3_O_4_ nanoparticles and were removed during the washing process. These results demonstrate that simple physical mixing is insufficient to achieve stable CNC–Fe_3_O_4_ association and highlight the necessity of an in situ coprecipitation strategy for robust attachment.

In this paper, S-CNC/Fe_3_O_4_ (Scheme 1c) and T-CNC/Fe_3_O_4_ (Scheme 1d) nanocomposites were synthesized in situ by coprecipitating Fe^2+^ and Fe^3+^ salts in the presence of CNCs at CNC:Fe_3_O_4_ mass ratios of 1:2 and 1:4. This approach is known to promote homogeneous nucleation and strong interfacial interactions between the CNC surfaces and the growing Fe_3_O_4_ nanoparticles, leading to uniform deposition and improved colloidal stability.^34^ A CNC:Fe_3_O_4_ ratio of 1:1 was investigated in a previous study on magnetic demulsification which showed that this ratio resulted in nonuniform surface coverage, with many CNCs remaining uncoated or only partially coated with Fe_3_O_4_.^20^ The characterization results of the in situ synthesized S-CNC/Fe_3_O_4_ and T-CNC/Fe_3_O_4_ nanocomposites are presented in the following sections.

### 3.2 Structural and Colloidal Properties

Transmission electron microscopy (TEM) images of the nanoparticles were acquired in bright field (BF)-scanning TEM (STEM) mode and are presented in Figure 1. High magnification STEM images of the samples are provided in Supporting Information, Figure S4. The S-CNCs (Figure 1a and Figure S4a) exhibited a rod-like morphology, with an average length of 140 ± 42 nm, which is consistent with our previous publication.^35^ The T-CNCs (Figure 1b and Figure S4b) also displayed a rod-like shape with an average length of 144 ± 41 nm, indicating that TEMPO oxidation did not alter the morphology of the cellulose nanocrystals, in agreement with the literature.^4^ The bare Fe_3_O_4_ nanoparticles (Figure 1c and Figure S4c) were quasi-spherical, with an average primary particle size of 21 ± 5 nm. However, in the absence of a surface coating, the bare Fe_3_O_4_ nanoparticles readily aggregate into clusters ranging from 100 to 500 nm. The EDS mapping of bare Fe_3_O_4_ NPs (Figure 1d) further confirmed the presence of Fe_3_O_4_ through characteristic Fe signals. Furthermore, STEM images of S-CNC/Fe_3_O_4_ (1:2 and 1:4) (Figures 1e–h and Figures S4d–e) confirmed the deposition of Fe_3_O_4_ nanoparticles on the S-CNC rod surfaces. The presence of S-CNC reduced Fe_3_O_4_ nanoparticle aggregation compared to bare magnetite, although some clustering of Fe_3_O_4_ remained visible in both samples. The S-CNC/Fe_3_O_4_ (1:4) sample displayed more extensive Fe_3_O_4_ clustering than the 1:2 counterpart, consistent with its higher Fe_3_O_4_ loading. The localization of Fe_3_O_4_ on the S-CNC surface was further corroborated by EDS mapping, where red signals correspond to Fe (Figures 1f,h). Similarly, STEM images of T-CNC/Fe_3_O_4_ (1:2 and 1:4), shown in Figures 1i,k and Figures S4f–g, confirmed Fe_3_O_4_ attachment to the T-CNC surfaces, with greater aggregation observed in the 1:4 sample. Notably, comparison of the two systems revealed that T-CNC/Fe_3_O_4_ exhibited more uniform and continuous surface coverage than S-CNC/Fe_3_O_4_, suggesting stronger Fe_3_O_4_ interactions with T-CNC, consistent with our DFT simulation results (Section 3.3). Additional STEM images of the S-CNC/Fe_3_O_4_ and T-CNC/Fe_3_O_4_ nanocomposites are provided in the Supporting Information (Figure S5), showing that T-CNC/Fe_3_O_4_ nanocomposites were more uniformly dispersed on the TEM grids compared to S-CNC/Fe_3_O_4_.

**Figure 1:**
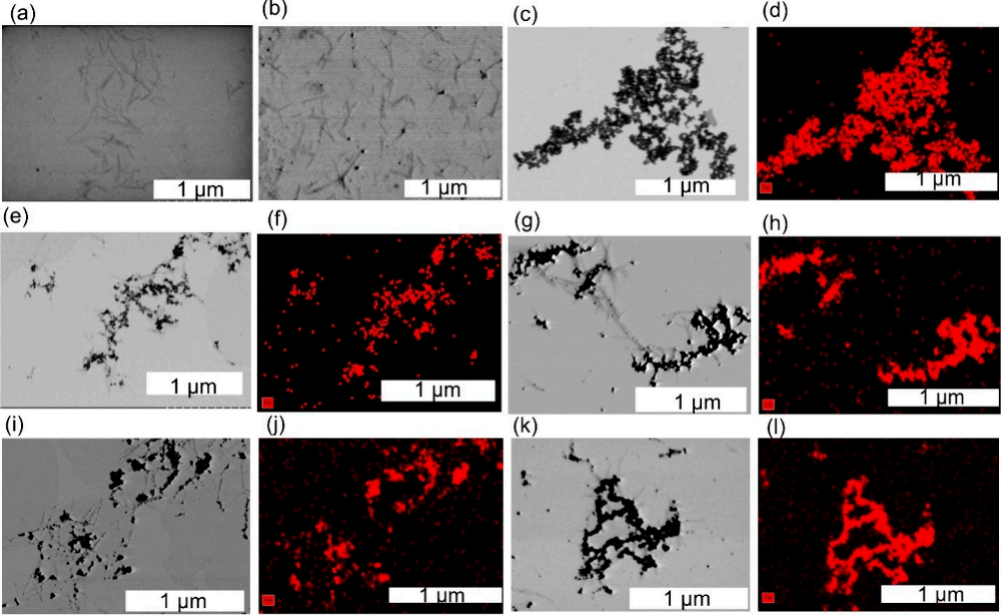
Bright-field STEM images and corresponding energy-dispersive X-ray (EDS) elemental maps for (a) S-CNC, (b) T-CNC, (c,d) bare Fe_3_O_4_; (e,f) S-CNC/Fe_3_O_4_ 1:2; (g,h) S-CNC/Fe_3_O_4_ 1:4; (i,j) T-CNC/Fe_3_O_4_ 1:2; and (k,l) T-CNC/Fe_3_O_4_ 1:4. STEM images show gray, rod-like CNC nanoparticles and the deposition of dark, spherical Fe_3_O_4_ nanoparticles on the CNC surface. EDS images show the presence of Fe in the CNC/Fe_3_O_4_ composites.

Zeta (ζ) potential and DLS particle size measurements across pH 3–10 are summarized in Figure 2a and Table 1. S-CNCs exhibited negative ζ potentials consistent with literature,^35^ originating from sulfate ester groups that provide electrostatic stabilization. S-CNC had ζ potentials below −32 mV, except at pH 3 where it was −26.8 ± 0.3 mV; T-CNCs showed nearly constant ζ potentials of −42 mV pH 5–10 due to combined sulfate and carboxylate groups from TEMPO oxidation. Both CNC types showed highly stable aqueous dispersions with particle sizes of ∼90 to106 nm. In contrast, bare Fe_3_O_4_ nanoparticles displayed positive ζ values at pH 3–5 and negative values at pH 7–10, with an isoelectric point around pH 6.03, consistent with the literature.^36,37^ The hydrodynamic diameter of bare Fe_3_O_4_ was ∼143 nm at pH 7. At more basic pH (9–10), higher surface charges produced smaller sizes (91–93 nm). Furthermore, S-CNC/Fe_3_O_4_ 1:2 showed ζ potentials below −30 mV at pH 5–10, with particle sizes of ∼182–210 nm, but aggregated heavily at pH 3, with sizes up to 3700 nm. Other nanocomposites, including S-CNC/Fe_3_O_4_ 1:2, T-CNC/Fe_3_O_4_ 1:2 and T-CNC/Fe_3_O_4_ 1:4, exhibited very similar trends. Overall, ζ potential and DLS results confirm that magnetic CNCs possess a higher negative surface charge than bare Fe_3_O_4_ nanoparticles across all pH values, indicating improved colloidal stability and supporting their suitability for various applications such as magnetic hyperthermia and antimicrobial treatment.

**Figure 2:**
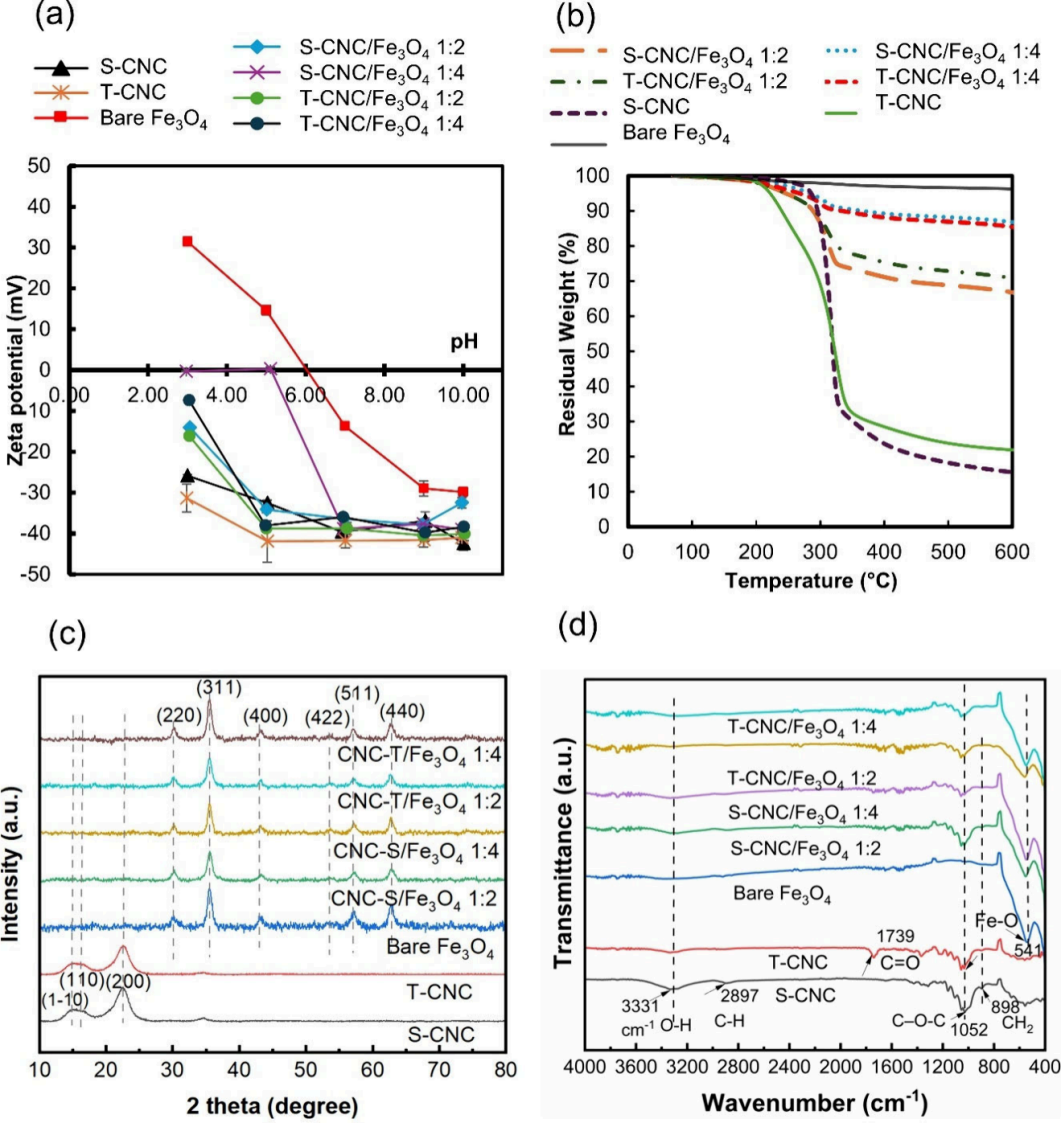
(a) Zeta potential measurements; (b) TGA thermograph; (c) X-ray diffraction pattern; (d) FT-IR spectra of S-CNC, T-CNC, bare Fe_3_O_4_, S-CNC/Fe_3_O_4_ 1:2, S-CNC/Fe_3_O_4_ 1:4, T-CNC/Fe_3_O_4_ 1:2, and T-CNC/Fe_3_O_4_ 1:4.

**Table 1:** Hydrodynamic Size of Various CNCs, Bare Fe_3_O_4_, and CNC/Fe_3_O_4_ Nanocomposites Measured at Different pH Values^a^

Sample Name	pH	Z-Average Size (nm)
**S-CNC**	3	104 ± 3
	5	92 ± 1
	7	107 ± 9
	9	91 ± 1
	10	91 ± 1
**T-CNC**	3	93 ± 1
	5	93 ± 1
	7	94 ± 1
	9	98 ± 1
	10	92 ± 1
**Bare Fe**_**3**_ **O**_**4**_ **NPs**	3	192 ± 5
	5	162 ± 6
	7	144 ± 18
	9	92 ± 9
	10	93 ± 8
**S-CNC/Fe**_**3**_ **O**_**4**_ 1:2	3	3715 ± 159
	5	211 ± 2
	7	210 ± 1
	9	200 ± 1
	10	183 ± 4
**S-CNC/Fe**_**3**_ **O**_**4**_ 1:4	3	2719 ± 210
	5	230 ± 3
	7	144 ± 2
	9	130 ± 1
	10	129 ± 1
**T-CNC/Fe**_**3**_ **O**_**4**_ 1:2	3	2618 ± 51
	5	210 ± 2
	7	221 ± 2
	9	183 ± 2
	10	190 ± 2
**T-CNC/Fe**_**3**_ **O**_**4**_ 1:4	3	3697 ± 112
	5	318 ± 4
	7	302 ± 7
	9	303 ± 4
	10	277 ± 2

a The uncertainty in pH measurement was within ± 0.05.

Thermogravimetric analysis (TGA) was performed to determine the actual magnetite (Fe_3_O_4_) content in magnetic cellulose nanocrystals. The Fe_3_O_4_ content was calculated based on the residual mass measured at 600 °C, representing the inorganic fraction. Figure 2b shows the TGA curves for neat CNCs, bare Fe_3_O_4_, and the magnetic CNCs, while the corresponding residual weights and calculated magnetite contents are shown in Supporting Information (Table S1). As expected, neat CNCs exhibited low residual weights, 15.5 ± 0.5% for S-CNC and 21.9 ± 0.5% for T-CNC, reflecting their predominantly organic composition.^38^ The significant weight loss observed between ∼250–400 °C corresponds to the thermal decomposition of CNC, associated with the breakdown of glycosidic bonds and depolymerization of cellulose chains.^20^ In contrast, bare Fe_3_O_4_ nanoparticles displayed a high residual mass of 96.3 ± 0.2%, consistent with their inorganic, thermally stable nature.^18,20^ The magnetic CNC nanocomposites showed increasing residual weights with higher Fe_3_O_4_ loading. For the 1:2 CNC:Fe_3_O_4_ formulations, S-CNC/Fe_3_O_4_ and T-CNC/Fe_3_O_4_ exhibited residual weights of 66.8 ± 0.5% and 70.9 ± 0.5%, corresponding to Fe_3_O_4_ contents of 63.5 ± 0.7% and 65.8 ± 0.7%, respectively. At the higher 1:4 CNC:Fe_3_O_4_ ratio, residual weights increased to 86.8 ± 0.5% for S-CNC/Fe_3_O_4_ and 85.4 ± 0.5% for T-CNC/Fe_3_O_4_, with corresponding Fe_3_O_4_ contents of 88.2 ± 0.7% and 85.4 ± 0.7%, respectively. These results confirm the successful incorporation of magnetite onto the CNC surfaces and demonstrate the strong correlation between the initial CNC:Fe_3_O_4_ feed ratio and the final Fe_3_O_4_ content in the nanocomposites.

The crystalline structure of the nanocomposites was characterized by X-ray diffraction (XRD), and the corresponding patterns are shown in Figure 2c. The XRD patterns of S-CNC and T-CNC exhibited identical diffraction peaks at 2θ = 14.84°, 16.24°, 22.4°, and 34.48°, corresponding to the (11̅0), (110), (200), and (004) crystallographic planes of cellulose I, confirming their semicrystalline nature.^39^ In contrast, bare Fe_3_O_4_ nanoparticles displayed distinct diffraction peaks at 30.08°, 35.48°, 43.36°, 53.56°, 57.28°, and 62.68°, which can be indexed to the (220), (311), (400), (422), (511), and (440) planes of the face-centered cubic spinel structure of magnetite (JCPDS 89–3854), consistent with previous reports.^40^ All magnetic CNC nanocomposites, regardless of CNC type or CNC:Fe_3_O_4_ ratio, exhibited the same set of peaks as bare Fe_3_O_4_, confirming the successful incorporation of crystalline iron oxide into the nanocomposite structure. It should be noted, however, that maghemite (γ-Fe_2_O_3_) phases typically show XRD peaks that overlap closely with those of magnetite, and XRD is not a sufficient technique to distinguish between the two. However, our magnetization results discussed later in this paper yield results consistent with iron oxide phases mostly made of magnetite.

The characteristic diffraction peaks of CNCs are absent in the magnetic nanocomposite samples (Figure S6b), mostly due to the extremely small X-ray attenuation coefficient of cellulose compared to Fe_3_O_4_. The linear attenuation coefficient (μ) for both, CNC and Fe_3_O_4_, were estimated using an online tool available at https://11bm.xray.aps.anl.gov/absorb/, developed by Argonne National Laboratory. For CNC, μ is approximately 11.62 cm^–1^ with a mass attenuation coefficient (MAC) of 7.088 cm^2^/g; while for Fe_3_O_4_, the values are 1154.3 cm^–1^ and 222.8 cm^2^/g, respectively. Thus, the intensity of the CNC peaks is reduced by a factor of 1/64 in the case of 1:2 nanocomposites and by 1/127 for 1:4 nanocomposites.^41^

Furthermore, the XRD peaks of the magnetic CNC nanocomposites were analyzed using the Williamson–Hall method to estimate the crystallite size and micro strain.^21^ The results showed that the crystallite size of bare Fe_3_O_4_ was ∼22 nm, consistent with literature,^42^ while the crystallite sizes of S-CNC/Fe_3_O_4_ (1:2), S-CNC/Fe_3_O_4_ (1:4), T-CNC/Fe_3_O_4_ (1:2), and T-CNC/Fe_3_O_4_ (1:4) were ∼9, ∼21, ∼8.5 nm, and ∼15 nm, respectively. Overall, nanocomposites with higher Fe_3_O_4_ loading (CNC:Fe_3_O_4_ = 1:4) tended to exhibit slightly larger crystallite sizes than those with lower Fe_3_O_4_ loading (CNC:Fe_3_O_4_ = 1:2). The smaller crystallite sizes observed in CNC/Fe_3_O_4_ nanocomposites compared to bare Fe_3_O_4_ can be attributed to the confinement and surface interactions provided by CNC, which limit crystal growth. At higher Fe_3_O_4_ loading (1:4), the reduced relative CNC surface area allows more unrestricted growth, leading to slightly larger crystallites. Moreover, the micro strain values obtained from Williamson–Hall analysis were 0.0062 for bare Fe_3_O_4_, 0.0158 for S-CNC/Fe_3_O_4_ (1:2), 0.0066 for S-CNC/Fe_3_O_4_ (1:4), 0.0162 for T-CNC/Fe_3_O_4_ (1:2), and 0.0093 for T-CNC/Fe_3_O_4_ (1:4). Overall, the magnetic CNC nanocomposites exhibit low lattice strain, with slightly higher values observed for the 1:2 composites compared to the 1:4 composites. This trend can be attributed to stronger particle–particle interfacial interactions and lattice distortions induced by CNC surface functional groups at lower Fe_3_O_4_ loading, whereas at higher Fe_3_O_4_ loading, the reduced relative effect of the CNC surface area results in lower lattice strain.

Fourier-transform infrared (FTIR) spectroscopy was employed to analyze the functional groups present in S-CNC, T-CNC, bare Fe_3_O_4_, and CNC/Fe_3_O_4_ nanocomposites (Figure 2d). The FTIR spectra of S-CNC showed characteristic peaks at ∼3331 cm^–1^ (O–H stretching), ∼2897 cm^–1^ (C–H stretching), ∼1654 cm^–1^ (O–H bending of absorbed water), ∼1428 cm^–1^ (−CH_2_ bending vibration), ∼1315 cm^–1^ (−CH_2_ wagging vibration), ∼1163 cm^–1^ (C–O–C asymmetric stretching), ∼1052 cm^–1^ (C–O–C pyranose ring vibration), and ∼898 cm^–1^ (C–H/CH_2_ bending vibration), consistent with existing literature.^20,39^ The T-CNC spectrum contained the same peaks with an additional band at 1743 cm^–1^, attributed to C=O stretching of carboxyl groups formed during TEMPO oxidation.^4,7^ This confirms the successful introduction of carboxyl functionalities on the CNC surface. Furthermore, the FTIR spectrum of bare Fe_3_O_4_ nanoparticles exhibited a prominent absorption band at 541 cm^–1^, corresponding to the Fe–O stretching vibration characteristic of magnetite.^43^ A more careful examination of the 2700–3600 cm^–1^ region shows that bare Fe_3_O_4_ exhibits an OH stretching peak at 3430 cm^–1^ (Supporting Information, Figure S6a), which is consistent with the literature.^44^ Magnetic CNC nanocomposites displayed FTIR spectra largely consistent with those of neat CNCs. These included characteristic peaks at 3333 cm^–1^ (O–H stretching), along with vibrations associated with C–H, C–O–C, and C–OH groups. Notably, in the magnetic CNCs, the O–H stretching band at 3333 cm^–1^ was broader and of lower intensity, and a weak shoulder was observed at 3450 cm^–1^, attributed to free −OH groups. These changes suggest surface interactions between the hydroxyl groups of CNCs and the Fe_3_O_4_ nanoparticles. Additionally, all magnetic CNC samples exhibited Fe–O absorption bands in the 540–560 cm^–1^ range, confirming the successful incorporation of magnetite onto the CNCs.^16^ Note that the C=O band decreased noticeably in intensity in the T-CNC/Fe_3_O_4_ samples, which may also indicate interaction between T-CNC and Fe_3_O_4_ through the carboxylate group. Furthermore, the interaction between CNC and Fe_3_O_4_ was examined in greater detail using XPS and density functional theory, as discussed in Section 3.3.

### 3.3 CNC–Fe_3_O_4_ Interactions

X-ray photoelectron spectroscopy (XPS) was performed to determine the chemical composition of the magnetic CNCs and to investigate possible interactions between the CNCs and Fe_3_O_4_ (Figure 3). The survey spectra of all samples were deconvoluted into the C 1s (B.E. 282–292 eV), O 1s (B.E. 528–538 eV), and Fe 2p (B.E. 705–735 eV) regions, as shown in Figure 3. The S-CNC and S-CNC/Fe_3_O_4_ (1:2 and 1:4) samples primarily exhibited three C 1s peaks: C1 (∼285 eV) corresponding to C–C/C–H linkages, C2 (∼286.7 eV) associated with C–O bonds in alcohols and ethers, and C3 (∼288 eV) attributed to O–C–O groups.^5^ In contrast, the T-CNC and T-CNC/Fe_3_O_4_ (1:2 and 1:4) nanocomposites displayed four C 1s peaks: C1 (∼285 eV) for C–C/C–H linkages, C2 (∼286.8 eV) for C–O in alcohols and ethers, C3 (∼288 eV) for O–C–O, and an additional C4 (∼289 eV) peak corresponding to O—C=O groups.^45^ The presence of this extra O—C=O peak in T-CNC and its nanocomposites confirms successful TEMPO-mediated oxidation of S-CNC to T-CNC.

**Figure 3:**
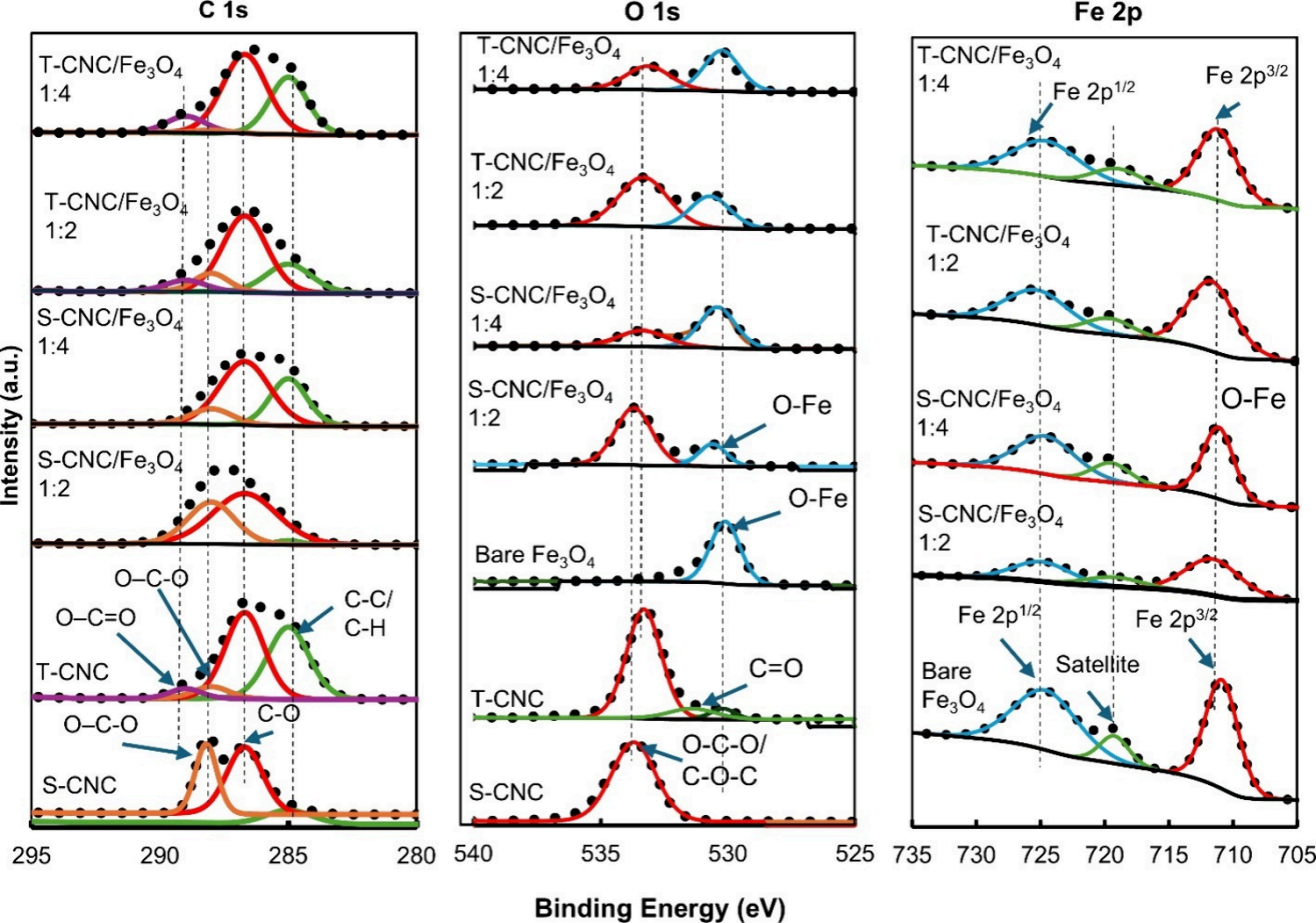
X-ray photoelectron spectroscopy (XPS) of S-CNC, T-CNC, bare Fe_3_O_4_, and various CNC/Fe_3_O_4_ nanocomposites.

In the O 1s spectra, the S-CNC sample exhibited a dominant peak at 533.8 eV, corresponding to O–C–O groups.^46^ In contrast, the S-CNC/Fe_3_O_4_ (1:2 and 1:4) nanocomposites displayed two main peaks: one at 533.8 eV (O–C–O) and an additional peak at 530.7 eV assigned to O–Fe bonds.^40,46^ The T-CNC sample showed two distinct peaks at 531.3 eV (C=O) and 533.4 eV (O–C–O), while the T-CNC/Fe_3_O_4_ (1:2 and 1:4) nanocomposites exhibited an additional peak at approximately 530.4 eV, corresponding to O–Fe bonds.^40^ The 533.4 eV (O–C–O) peak of T-CNC and T-CNC/Fe_3_O_4_ appears at a lower binding energy than that of S-CNC,^47^ which is likely associated with formation of carboxylate groups during TEMPO oxidation that increases local electron density and allows some delocalization over the O–C–O unit. This increased electron density decreases the effective binding energy of the O 1s electrons, although minor contributions from different charging/reference effects cannot be excluded. For comparison, bare Fe_3_O_4_ nanoparticles showed a single O 1s peak at 530.1 eV, characteristic of O–Fe bonding. The higher binding energy of the O–Fe peak in all magnetic CNC nanocomposites compared to bare Fe_3_O_4_ indicates a more positive environment in the oxygen atoms bonded to Fe, consistent with the DFT-predicted shifts from the Bader charge analysis after adsorption of the Fe_3_O_4_ cluster (discussed in the following DFT simulation section).

The Fe 2p XPS spectra of bare Fe_3_O_4_ show a Fe 2p_3_/_2_ peak at ∼711.0 eV, consistent with the mixed Fe^2+^/Fe^3+^ oxidation states in magnetite,^48^ along with a satellite at 719 eV.^48^ The spectral line shape resembles Fe_2_O_3_ more than Fe_3_O_4_,^40^ suggesting partial surface oxidation postsynthesis, in agreement with previous reports that nonstoichiometric Fe_3_O_4_ can produce a Fe^3+^ satellite due to the loss of the Fe^2+^ satellite from FeO.^49^ In the CNC/Fe_3_O_4_ nanocomposites, the Fe 2p_3_/_2_ peaks shifted slightly to higher binding energies (711.2–712.0 eV), likely due to electronic interactions between surface Fe atoms and the oxygen-containing groups on the CNCs, which slightly reduce the local electron density around the iron centers; however, the shift is only appreciable for the 1:2 mass ratio. The Fe 2p_1_/_2_ peak at ∼724.8 eV and the satellite at ∼719.3 eV remain almost unchanged, confirming that the Fe oxidation states remain consistent with magnetite and are retained.

To rationalize the experimental characterizations of the binding nature between T-CNC and magnetite, and to provide deeper insight into the driving force behind the colloidal stability of T-CNC/Fe_3_O_4_, we carried out periodic DFT calculations. In this study we considered the minimal (Fe_3_O_4_)_n_ cluster with *n* = 1. The magnetite cluster model is based on the Mejia-Olvera study^50^ and the description of the cluster is in Figure 4c. The minimal magnetite cluster limits the description of global electronic structure and magnetization due to its finite size and the lack of long-range interactions. However, our intent is to qualitatively predict the local charge/spin polarization of the magnetite/CNC interface. Many studies^51,52^ also reported interfacial studies using the minimal magnetite cluster approach. First, we investigated the adsorption of the cluster on seven distinct sites of the S-CNC/T-CNC (100) surface, based on our FTIR and XPS observations. Those sites are the bridged oxygen connecting the 6-membered rings (O1), oxygens in the secondary hydroxyl group (O2 and O3), oxygen in the 6-membered ring (O5), oxygen in the primary alcohol (O6), oxygen in the carboxyl group (Ot), and the carbon of the −CH_2_OH (C6) group as described in Figure 4. All the computed adsorption energies per (Fe_3_O_4_) formula unit and magnetic properties are tabulated in Table S4. Our DFT calculations found that the pristine CNC (100) surface favors the cluster (bridged bidentate mode) when two Fe atoms are bonded with two oxygen atoms (O1 and O6) of the surface, with an adsorption energy of −1.88 eV. The adsorption site O5 favors the adsorption with −1.53 eV, the adsorption site O1 and O5 bonded with one Fe atom (bidentate mode) with −1.11 eV, and the adsorption energy (−0.39 eV) is least favorable on top of C6 which acts like a physisorption mode. In the case of T-CNC, the *E*_ads_ is more negative for the O5/Ot adsorption sites (bidentate mode), where oxygen from the −COOH group stabilizes the cluster. The O2 adsorption site is less favorable for T-CNC, with an *E*_ads_ of only −0.82 eV, where adsorption at Ot reaches −1.93 eV.^53,54^ Also, the spin density plot of T-CNC/O1/O6–Fe, showing all the spin up characteristics, can explain the increase in magnetic moment, although it shows a moderate adsorption energy. The most stable configuration of the T-CNC/(Fe_3_O_4_)_*n*=1_ composite exhibits the largest negative surface magnetization (−0.05 μ_B_) of the oxygen (Ot) in the carboxyl group and −OH group. However, the effect of the cluster is minimal on pristine CNC. All the composite systems of T-CNC are shown in Figure 5(a–d).

**Figure 4:**
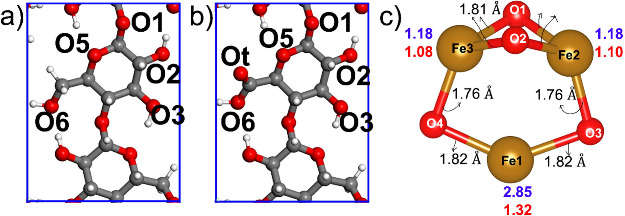
(a) Adsorption sites on pristine S-CNC (100), (b) on T-CNC (100), and (c) description of the magnetite cluster with bond length (black), Bader charge (red) and magnetic moment (blue). The total magnetic moment is 5.49 μ_B_. Color code: red, gray, and white indicate oxygen, carbon and hydrogen atoms, respectively. Blue line indicates the simulation cell. The O1 of one unit is the O4 of the next, and we label it by O1.

**Figure 5:**
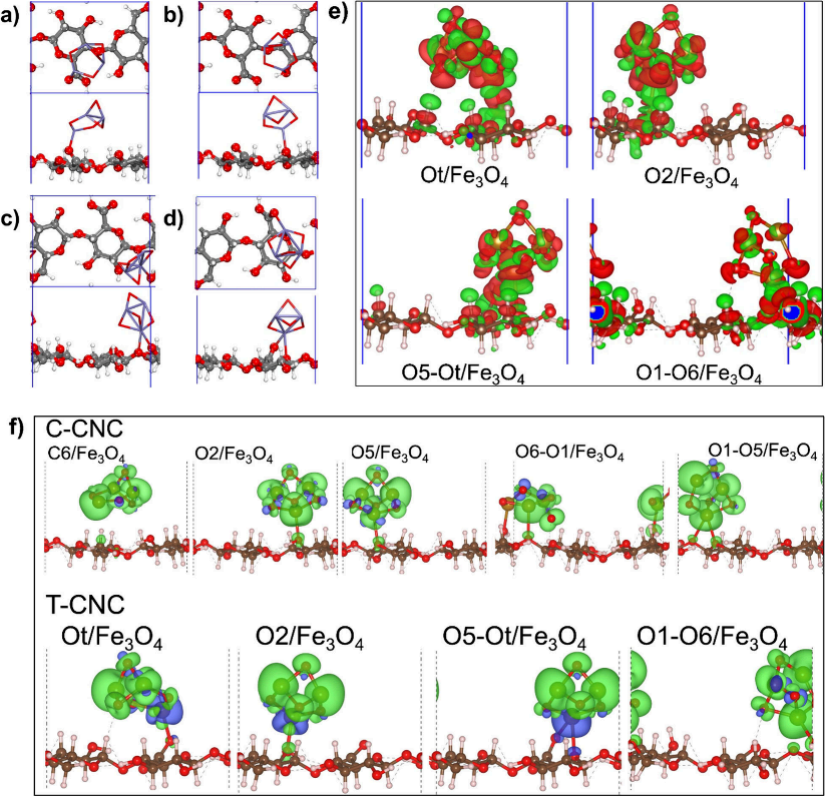
Optimized geometry of T-CNC/Fe_3_O_4_ with their top and side view. (a) T-CNC/Ot-Fe, (b) T-CNC/O2–Fe, (c) T-CNC/O1/O6–Fe, (d) T-CNC/O5/Ot-Fe, (e) charge density differences of the systems (a) to (d), and (f) spin density of the different systems of S-CNC and T-CNC. Red color indicates depletion of charge while green indicates accumulation of charge (charge density plot), while green and blue indicates positive and negative spin moment, respectively (spin density plot). The isovalue of charge density and spin density is used 0.002 e/Bohr^3^ and 0.004 e/Bohr^3^, respectively.

We further analyzed the bonding nature (Fe_3_O_4_)_*n*=1_ on T-CNC by employing multiple complementary analyses, including Bader charge analysis,^55^ charge density difference (CDD) maps, partial density of states (PDOS), and integrated COHP (ICOHP). Through COHP,^56^ the strength of interfacial covalent bonds was quantitatively evaluated. By definition, the most negative ICOHP value indicates the strongest covalent interaction.

The Bader charge analysis of the S-CNC or T-CNC/(Fe_3_O_4_)_*n*=1_composite systems is described in Table S5. The analysis outlined in all cases, the bonded Fe atom showing a positive charge and the cellulose O atoms gaining electrons. The results support the idea that all adsorption is stabilized mainly by electrostatic interactions rather than hydrogen bonding. Moreover, the CDD plot indicates that charge accumulation occurs at the Fe–O interfacial bonds in all cases, including CNC and T-CNC (Figure 5e). Bader charge analysis of the (Fe_3_O_4_)_*n*=1_ cluster before and after the adsorption revealed that the net charge of the adsorbed cluster is positive or neutral (Supporting Information, Table S6). However, there is a charge redistribution among the atoms in the adsorbed magnetite. In all cases (except T-CNC/Ot-Fe), the net charge of oxygen atoms is less negative (i.e., shows a positive charge shift) than in the preadsorbed ones. This result is consistent with the XPS data, which indicated a shift toward higher binding energies for the O–Fe component in the O 1s signal. It is noted that the CDD plot and the net charge of the adsorbed cluster indicate there is a charge transfer from the cluster to the CNC/T-CNC surface. In the case of iron, the net charge shows a reduction in positive charge for most adsorption sites, contrary to what was expected based on the XPS results, which indicated a small, but noticeable shift toward high binding energy for the composites with 1:2 mass ratio. However, there is a net positive change in the total Bader charge of the Fe_3_O_4_ cluster after adsorption for the most stable configurations (lower *E*_ads_). We believe that thermal fluctuations combined with a higher net positive charge of the Fe_3_O_4_ may lead to the observed positive shift in the binding energy of Fe 2p_3_/_2_ peak.

The computed PDOS of the S-CNC and T-CNC (Supporting Information, Figure S1c) confirms that the valence band maxima (VBM) are derived primarily from oxygen, while the conduction band minima (CBM) are derived primarily from carbon. In the case of T-CNC, a new state was observed at 4.1 eV, corresponding to the carboxyl (−COOH) group, as shown in Figure S1c. The PDOS of pure magnetite and the most stable T-CNC/Fe_3_O_4_ composite system are shown in Figure S7. The VBM and CBM of the T-CNC shift upon magnetite adsorption, and the gap is entirely occupied by the DOS of magnetite, as shown in Figure S7­(a, c) and Figure S1c. The PDOS of T-CNC/Fe_3_O_4_ shows the presence of a spin-polarized peak near the Fermi level, which indicates surface magnetization originating from interfacial iron sites (Figure S7c).^57^ In addition, we can observe that the new peak is arising in the PDOS of the adsorbed system relative to the pure magnetite, as shown in Figure S7­(b, d), suggesting that the new bonds are formed due to the electrostatic interaction. The computed ICOHP value is used to compare the bond strength of each Fe–O bond. The ICOHP value of the Fe-Ot bond (2.02 Å) is −3.44 eV, which suggests the formation of a strong covalent bond. The magnetic moment calculation reveals that the increased magnetic moment of the surface oxygen at the −COOH group dictates the stability of magnetite adsorption on T-CNC. Conversely, the ICOHP value, shown in Figure S7 (e,f), for the bidentate T-CNC/O5/Ot-Fe system has two different values: the Fe–O5 bond (2.46 Å) is −1.81 eV, and the Fe-Ot bond (2.18 Å) is −2.71 eV. The total ICOHP value for the O5/Ot site is −4.52 eV, which also suggests a strong bond, consistent with the most negative *E*_ads_ and highest magnetization of the bonded surface atom. By accounting for all the analyses of interfacial properties, such as interfacial charge density, electronic interactions, and adsorption energy, one can provide a microscopic explanation for the improved dispersion and magnetic response observed in the magnetic hyperthermia performance.

### 3.4 Magnetic Properties

The magnetic properties of the synthesized S-CNC/Fe_3_O_4_ and T-CNC/Fe_3_O_4_ nanocomposites were evaluated using a vibrating sample magnetometer (VSM) and compared with bare Fe_3_O_4_ nanoparticles. The magnetization curves (Figure 6a) were normalized per gram of sample first. The bare Fe_3_O_4_ nanoparticles exhibited the highest saturation magnetization as indicated in Table S7, followed by S-CNC/Fe_3_O_4_ (1:4), T-CNC/Fe_3_O_4_ (1:4), S-CNC/Fe_3_O_4_ (1:2), and T-CNC/Fe_3_O_4_ (1:2). In both systems, increasing the Fe_3_O_4_ content increased the saturation magnetization, confirming successful immobilization of Fe_3_O_4_ nanoparticles on the CNC surfaces. The hybrid magnetic CNC nanocomposites exhibited superparamagnetic behavior similar to bare Fe_3_O_4_, consistent with previous reports in the literature.^58^ The saturation magnetization of the samples was also normalized to the amount of magnetite present in each composite (Figure 6b and Table S7). The highest normalized saturation magnetization among the nanocomposite samples was for S-CNC/Fe_3_O_4_ (1:2), 77.0 ± 1.0 emu g^–1^ of magnetite, and S-CNC/Fe_3_O_4_ (1:4), 77.6 ± 0.8 emu g^–1^ of magnetite, values comparable to that of bare Fe_3_O_4_ nanoparticles (82.5 emu g^–1^). In contrast, T-CNC/Fe_3_O_4_ (1:2) and (1:4) showed lower values of 66.5 and 60.1 emu g^–1^ of magnetite, respectively. Overall, T-CNC/Fe_3_O_4_ composites displayed reduced magnetization compared to their S-CNC/Fe_3_O_4_ counterparts, which aligns with our DFT results (Section 3.3). This reduction may stem from strong coordination of the carboxylate groups (COO^–^) from TEMPO-oxidized CNC at the Fe_3_O_4_ surface. The carboxylate binding likely induces surface restructuring or disorder, which decreases the net magnetic moment.^59^

**Figure 6:**
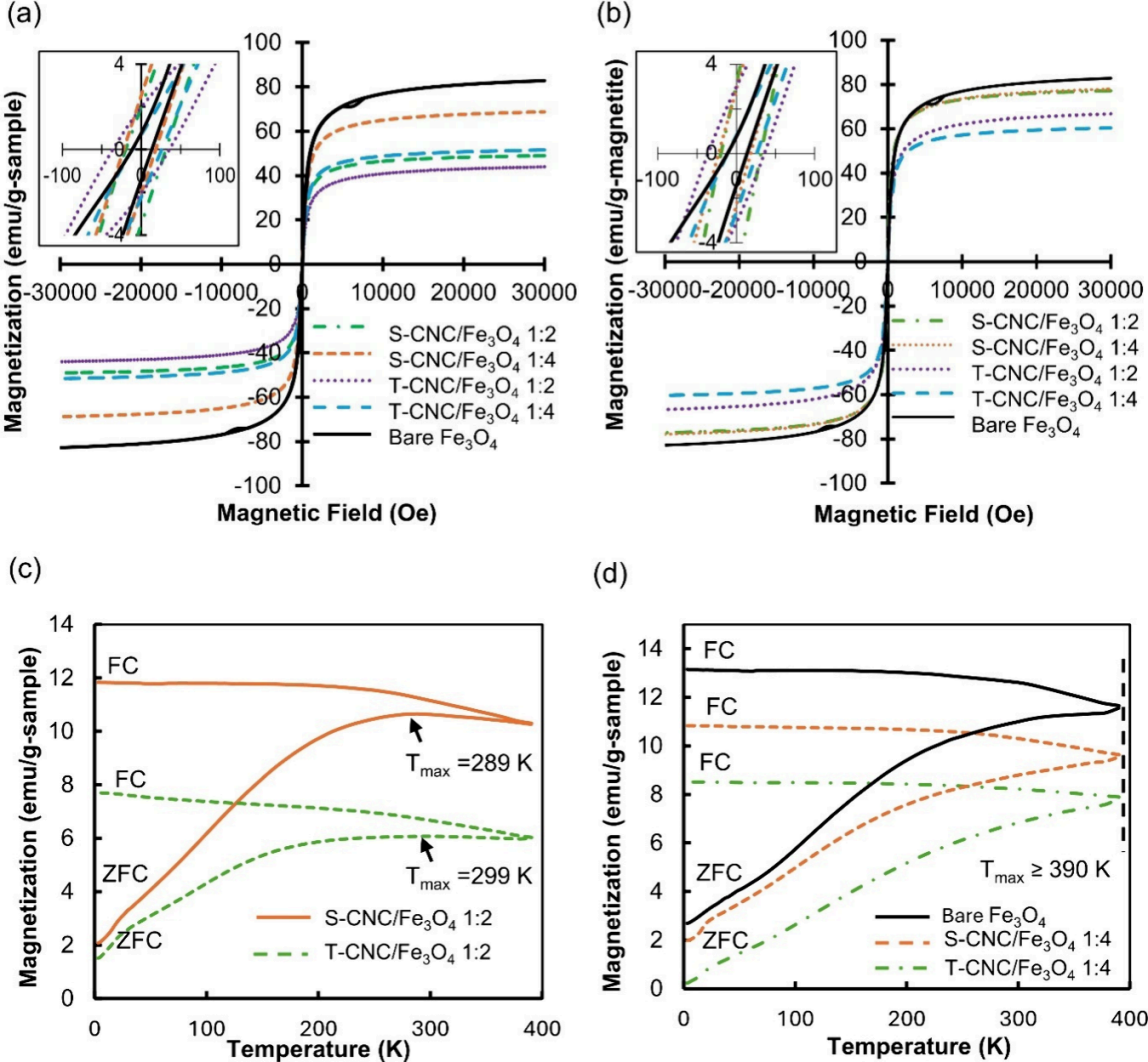
Magnetic properties of S-CNC/Fe_3_O_4_, T-CNC/Fe_3_O_4_, and bare Fe_3_O_4_: (a) magnetization per gram of sample, (b) magnetization per gram of magnetite, (c) FC (100 Oe) and ZFC curves of S-CNC/Fe_3_O_4_ 1:2 and T-CNC/Fe_3_O_4_ 1:2, and (d) FC (100 Oe) and ZFC curves of bare Fe_3_O_4_, S-CNC/Fe_3_O_4_ 1:4, and T-CNC/Fe_3_O_4_ 1:4. The insets of Figures 6a and 6b show the *M*_r_ and Hc points on the VSM curves.

Furthermore, the remanence (*M*_r_) and coercivity (Hc) values were extracted from the VSM curves, as shown in the insets of Figures 6a and 6b, and are summarized in Table S7. All magnetic CNC composites, including bare Fe_3_O_4_, exhibited very low *M*_r_ values (<2.5 emu/g) and low Hc values (<34 Oe), indicating that they possess the characteristic behavior of superparamagnetic nanoparticles. These results confirm that the incorporation of CNC does not compromise the superparamagnetic nature of Fe_3_O_4_, which is crucial for applications such as magnetic hyperthermia and targeted delivery.

Field-cooled (FC) at 100 Oe and zero-field-cooled (ZFC) magnetization curves for bare Fe_3_O_4_ nanoparticles and the CNC/Fe_3_O_4_ nanocomposites were recorded from 3 to 390 K (Figures 6c,d). The ZFC curve of bare Fe_3_O_4_ showed a monotonic increase up to the highest measured temperature (390 K), consistent with strongly interacting or aggregated magnetite nanoparticles.^60^ The S-CNC/Fe_3_O_4_ (1:2) and T-CNC/Fe_3_O_4_ (1:2) samples display clear ZFC maxima at approximately 289 and 299 K. Such large ZFC maxima values are generally attributed to increased magnetic polydispersity, particle volume, enhanced anisotropy, strong interparticle interactions, and possible core–shell or collective effects.^61^ Blocking temperature obtained from the conventional ZFC maxima may not accurately reflect the behavior of aggregated particles or samples with a broad size distribution. In this study, we determined the blocking temperature using the temperature derivative of the difference between the ZFC and FC curves, as proposed by Micha et al. and validated by Bruvera et al.^62,63^ The blocking temperature (T_B_) of all magnetic CNC nanocomposites, including bare Fe_3_O_4_, was found to be 112.1 K, except for T-CNC/Fe_3_O_4_ (1:2), which exhibited a T_B_ of 99.5 K (shown in Table S7). The blocking temperature of the nanocomposites (T_B_ ∼ 112 K) indicates that the magnetic moments of the CNC/Fe_3_O_4_ nanoparticles have enough thermal energy to orient in response to an external magnetic field above this temperature, confirming their superparamagnetic behavior at room temperature. This ensures negligible remanence and coercivity, which is desirable for applications such as magnetic hyperthermia and targeted delivery. Overall, the superparamagnetic behavior of the CNC/Fe_3_O_4_ composites, indicated by their Ms and T_B_ values, is favorable for magnetic hyperthermia applications. Higher Ms is often related to an enhanced heating efficiency, while T_B_ values below body temperature ensure rapid magnetic relaxation, both of which contribute to increased SAR under an alternating magnetic field.

### 3.5 Magnetic Hyperthermia and Specific Absorption Rate (SAR)

Magnetic hyperthermia is an important functional evaluation of magnetic nanomaterials, particularly for biomedical applications such as cancer therapy, targeted drug delivery, and localized heating, as it assesses their ability to convert electromagnetic energy into heat under an alternating magnetic field.^18,64^ Figures 7a,b,c show the heating profiles of S-CNC/Fe_3_O_4_, T-CNC/Fe_3_O_4_ and bare magnetite dispersed in water, recorded under alternating magnetic fields of 6.3 ± 0.1, 18.5 ± 0.2, and 30.4 ± 0.9 kA/m for 600 s. At the lowest field (6.3 ± 0.1 kA/m), all samples showed only a modest temperature increase of about 3–6 °C, indicating limited magnetic energy loss at low field amplitudes. As the magnetic field strength increased to 18.5 ± 0.2 kA/m, heating increased markedly, with the maximum temperature rising to 60–70 °C depending on the sample. The most pronounced heating was observed at the highest field (30.4 ± 0.9 kA/m), where the temperature exceeded 90 °C for S-CNC/Fe_3_O_4_ (1:4) and T-CNC/Fe_3_O_4_ (1:4), demonstrating a strong magnetic responsiveness and efficient energy dissipation. In contrast, nanocomposites with lower Fe_3_O_4_ loading (1:2) exhibited comparatively lower heating, consistent with reduced magnetic content and weaker interparticle interactions.

**Figure 7:**
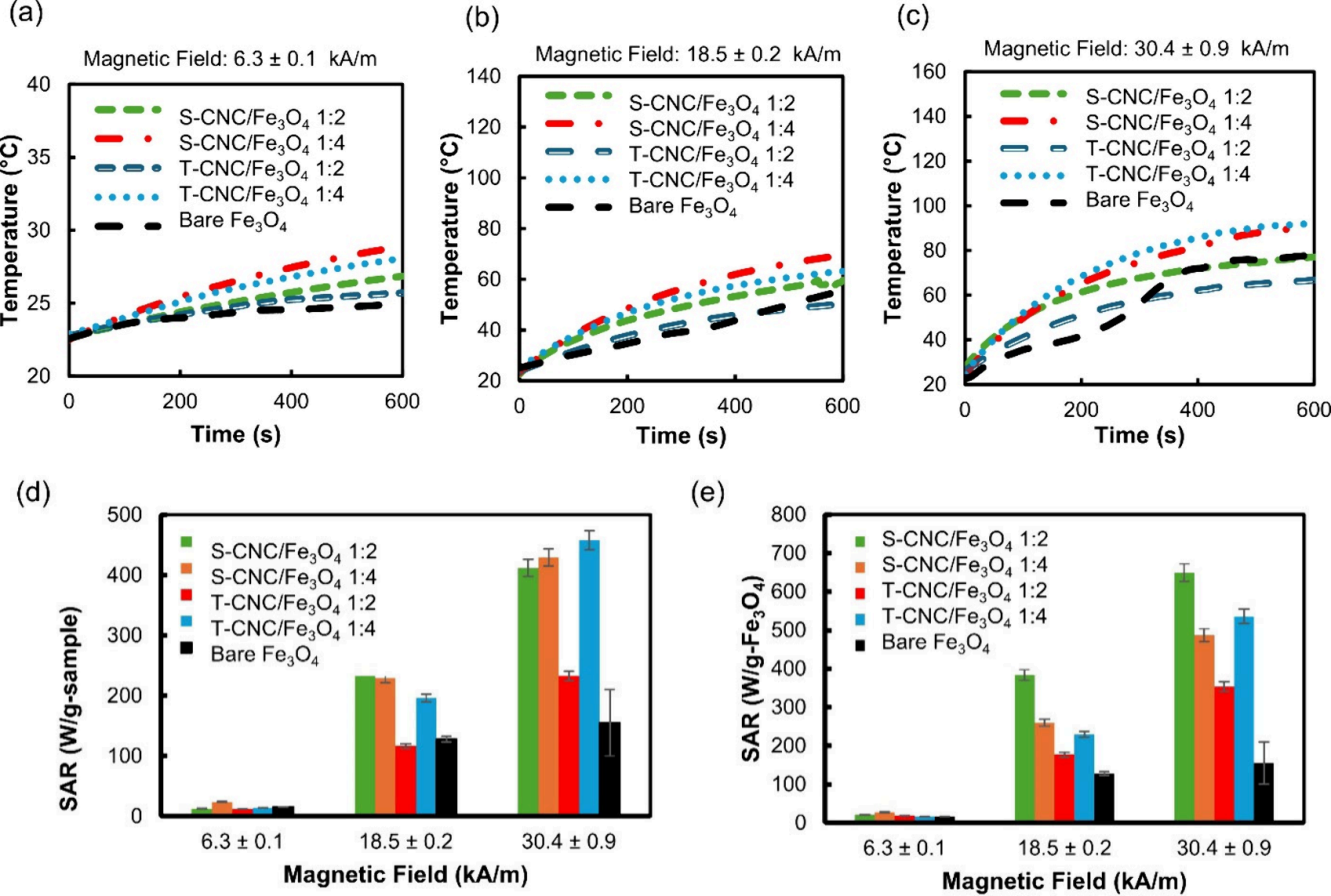
Magnetic hyperthermia heating profiles of magnetic nanoparticle samples at 0.3 wt % concentration (in water) under a 155 ± 4 kHz alternating magnetic field with amplitudes of (a) 6.3 ± 0.1 kA/m, (b) 18.5 ± 0.2 kA/m, and (c) 30.4 ± 0.9 kA/m. Specific absorption rate (SAR) of the magnetic CNC samples at different magnetic fields: (d) SAR per gram of sample and (e) SAR normalized per gram of magnetite in the sample.

The specific absorption rate (SAR) values of the four magnetic CNC/Fe_3_O_4_ nanocomposites along with bare Fe_3_O_4_ were evaluated under the same alternating magnetic fields, as shown in Figures 7d,e. At 6.3 ± 0.1 kA/m, SAR values per gram of sample ranged from approximately 12.8 W/g for S-CNC/Fe_3_O_4_ (1:2) to 23.5 W/g for S-CNC/Fe_3_O_4_ (1:4), with T-CNC/Fe_3_O_4_ (1:2) and T-CNC/Fe_3_O_4_ (1:4) exhibiting intermediate values. As the magnetic field strength increased to 18.5 ± 0.2 kA/m and 30.4 ± 0.9 kA/m, SAR values rose significantly, with S-CNC/Fe_3_O_4_ (1:4) reaching up to 429 W/g at 30.4 ± 0.9 kA/m. This field-dependent enhancement is characteristic of superparamagnetic systems, where higher magnetic amplitudes promote greater Néel and Brownian relaxation losses, thereby increasing heat dissipation.^65^ Normalization of SAR values per gram of magnetite revealed that S-CNC/Fe_3_O_4_ (1:2) exhibited the highest intrinsic heating efficiency at all field strengths, suggesting that moderate magnetite content combined with good dispersion provides optimal magnetic loss.^65^ Lanier et al. (2019) investigated 31 different commercial magnetic nanoparticles for magnetic fluid hyperthermia, examining how their intrinsic properties, such as iron content, hydrodynamic diameter, magnetic diameter, saturation magnetization, core size, and thickness of the magnetically dead layer, influence their heating performance and SAR.^19^ Among these parameters, the iron content, mass magnetization, and thickness of the magnetically dead layer showed significant positive correlations with the SAR values, whereas the other properties exhibited only low to moderate correlations with SAR.^19^ In our study, magnetic CNC nanocomposites with higher magnetite content exhibited greater heating efficiency and higher SAR values, consistent with the findings of Lanier et al. (2019).^19^ However, when the SAR values were normalized per gram of magnetite, the S-CNC/Fe_3_O_4_ (1:2) sample displayed the highest intrinsic heating efficiency (SAR 649 W/g-Fe_3_O_4_). This behavior can be attributed to its high saturation magnetization (77.26 emu/g–magnetite), combined with reduced Fe_3_O_4_ nanoparticle aggregation. The S-CNC/Fe_3_O_4_ (1:4) nanocomposite also had a high magnetization of 77.90 emu/g magnetite; however, the ZFC/FC curves and the TEM images indicate higher Fe_3_O_4_ aggregation compared to the 1:2 ratio. The reduced aggregation at the 1:2 ratio contributes to shorter Brownian and Néel relaxation times under an alternating magnetic field, thus these composites can more quickly align their magnetic moments with the external field direction, leading to larger SAR.^66^ Despite having lower saturation magnetization than their S-CNC/Fe_3_O_4_ counterparts, the T-CNC/Fe_3_O_4_ (1:4) nanocomposites exhibited substantial heating rates and SAR values, comparable to the S-CNC/Fe_3_O_4_ samples. This performance, combined with improved colloidal stability and more uniform Fe_3_O_4_ distribution on the CNC matrix, makes the T-CNC/Fe_3_O_4_ (1:4) nanocomposites promising candidates for magnetic hyperthermia applications. Overall, CNC/Fe_3_O_4_ nanocomposites exhibit effective heating under safe magnetic hyperthermia conditions, with the applied field and frequency (155 ± 4 kHz, as used in this study) remaining within Hergt’s clinical limits (*H* × *f* ≤ 5 × 10^9^ A·m^–1^·s^–1^).^33^

Interestingly, bare Fe_3_O_4_ nanoparticles exhibited lower SAR and a smaller temperature rise than S-CNC/Fe_3_O_4_ (1:2 and 1:4) and T-CNC/Fe_3_O_4_ (1:4) and were only slightly higher than T-CNC/Fe_3_O_4_ (1:2) at 6.3 ± 0.1 and 18.5 ± 0.2 kA/m magnetic field strength. At 30.4 ± 0.9 kA/m, bare Fe_3_O_4_ nanoparticles exhibited lower SAR and a smaller temperature rise than all CNC/Fe_3_O_4_ composites. The reduced SAR and heating efficiency of bare Fe_3_O_4_ are likely attributable to a variety of factors including particle aggregation and strong dipole–dipole interactions, which reduces magnetic relaxation speed and energy dissipation under an alternating magnetic field. This behavior was observed in the irregular heating profile of bare magnetite nanoparticles at 30.4 ± 0.9 kA/m (Figure 7c and Figure S8). The profile starts with a low slope, which increases after 200 s, and finally levels off near 400 s. The sudden slope growth could be caused by a period of chain aggregation of the magnetic nanoparticles in the presence of the magnetic field leading to increased anisotropy and faster heating. Additional aggregation into clusters and eventual particle precipitation then leads to a slow heating region with a reduced slope. This behavior is likely related to the low surface charge of the bare magnetite nanoparticles, which reduces their colloidal stability compared to CNC/Fe_3_O_4_ composites at neutral pH, where the magnetic hyperthermia experiments were performed. These results highlight the importance of stabilizing magnetite nanoparticles with stabilizers or surface coatings to maintain uniform heating behavior.^67^ In contrast, deposition of Fe_3_O_4_ on the CNC surface increases the Fe_3_O_4_ interparticle separation and improves colloidal stability, helping promote more efficient Néel and Brownian relaxation. In addition, the geometric anisotropy of the CNC/Fe_3_O_4_ nanocomposites is expected to correlate to a larger magnetic anisotropy constant compared to bare Fe_3_O_4_. Thus, the higher anisotropy of the nanocomposites leads to a higher amount of energy required to align the moments in the direction of the magnetic field, which also contributes to increasing energy losses and their SAR value. These effects, collectively, lead to higher SAR and improved heating performance in CNC/Fe_3_O_4_ composites. Overall, the results confirm that magnetic field strength, nanoparticle loading, anisotropy and reduced aggregation play important roles in determining the heating efficiency of CNC-based magnetic nanocomposites, underscoring their potential utility in magnetic hyperthermia and other magnetically driven thermal applications, such as magneto-responsive dispersions, self-standing films, and magnetic devices.^68^

### 3.6 Cytotoxicity

Cytotoxicity assessment is a critical step in evaluating the biocompatibility of nanomaterials for biomedical applications. In this study, RAW 264.7 macrophages were exposed to cellulose nanocrystals (S-CNC, T-CNC) and their magnetic composites (S-CNC/Fe_3_O_4_, T-CNC/Fe_3_O_4_) at three different concentrations (20, 100, and 500 μg/mL) for 24 h, and cell viability was assessed using both LDH and MTT assays (Figure 8). Lactate dehydrogenase (LDH) activity in the culture supernatant, which is a proxy for cellular membrane damage,^69^ remained low across all nanoparticle treatments (Figure 8a). More specifically, treatments with S-CNC, T-CNC, and composites at 500 μg/mL resulted in significantly lower LDH activity values. Because the samples for LDH activity assay were taken from the very top of the culture supernatant, the lower LDH activities were unlikely to be attributed by direct nanoparticle interference with the assay. Instead, it is possible that at such a high concentration, the cellulose nanocrystals provided protective effects from LDH leaking into the culture media during the 24 h of incubation. This hypothesis is supported by the observation that at the same 20 μg/mL of nanoparticle concentration, CNC only samples, which contain relatively more CNC than composites, exhibited a significant effect compared to no nanoparticle controls. The specific molecular mechanism, such as the role of phagocytosis, underlying this response remains to be determined. Nevertheless, it is clear that at 20 and 100 μg/mL, we did not detect any effects from any of the composite nanoparticles on LDH activity in RAW 264.7 macrophages.

**Figure 8:**
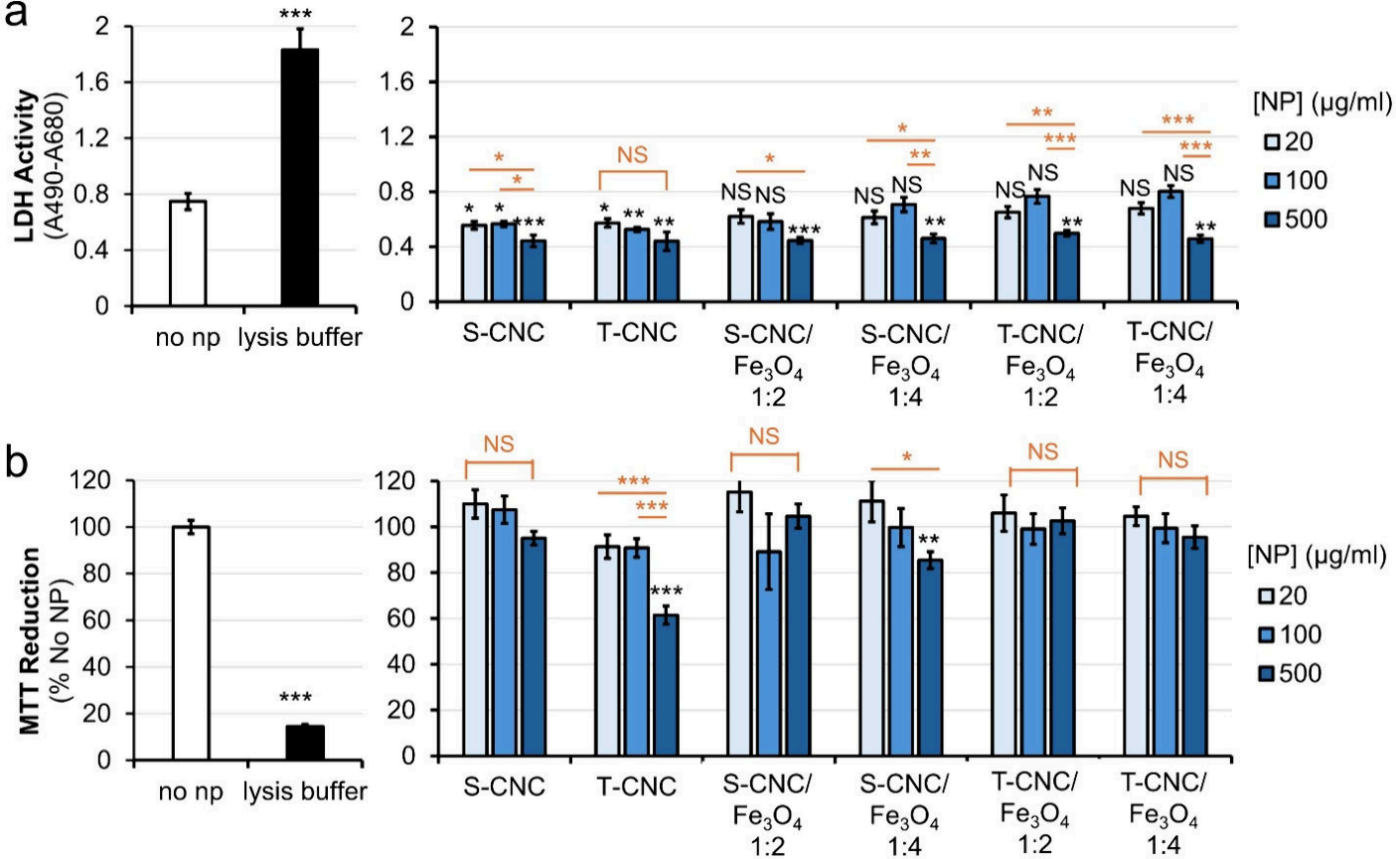
Toxicity measurements of the synthesized particles. (a) Lactate dehydrogenase (LDH) activity and (b) tetrazolium (MTT) reduction of RAW 264.7 macrophages in response to 24 h of nanoparticle treatments. Averages of 8 replicates across two independent experiments were plotted with error bars representing standard errors of the means (SEM). Significant differences between the no nanoparticles and other samples were marked by black asterisks. Significant differences between samples with 20 μg/mL nanoparticles and other concentrations within the same types of nanoparticles were marked by orange asterisks. The number of asterisks represents p values based on the following: *, *p* ≤ 0.05; **, *p* ≤ 0.01; ***, *p* ≤ 0.001. “NS” denotes no significant difference (*p* > 0.05).

MTT reduction assays, which monitor intracellular enzymatic activities, were also performed to further investigate the effects of the particles on adherent RAW 264.7 and to provide additional support for LDH activity results. By setting the no nanoparticle samples as 100%, we observed that while lysis buffer significantly decreased MTT reduction activity, only T-CNC at the 500 μg/mL concentration but not S-CNC or T-CNC at 20 or 100 μg/mL exhibited an inhibitory effect on the MTT reduction activity (Figure 8b). It is important to note that a centrifugation step was performed to remove nanoparticles prior to MTT measurement, so it is unlikely that the decrease in MTT reduction activity was a result of direct interference in absorbance reading by T-CNC. Considering that a reduced LDH activity is also observed in samples treated with 500 μg/mL T-CNC, it is likely that at this high concentration, T-CNC potentially reduce the growth of RAW 264.7 macrophages, thereby leading to less mitochondrial MTT reduction, without causing lysis. RAW 264.7 cells are an immortalized murine peritoneal macrophage cell line widely used in host–pathogen research. The different effects from S-CNC and T-CNC on RAW 264.7 macrophages might provide a unique approach to study metabolic and cell permeability regulation in response to CNCs.

Compared to no nanoparticle control samples, treatments with S-CNC/Fe_3_O_4_ 1:4 at 500 μg/mL was the only composite where a significant decrease in MTT reduction activity was observed (Figure 8b). All other composites, compared to no nanoparticle controls, showed no significant effects on MTT activity across the 3 concentrations tested. These observations again highlight the different responses in RAW 264.7 macrophages to S-CNCs and T-CNCs, further arguing for future investigations to determine the cellular mechanisms allowing the macrophages to distinguish and respond differently to these nanoparticles. Nevertheless, at 20 and 100 μg/mL, all composites showed no detectable effects on the MTT reduction activity in macrophages.

## 4 Conclusions

Magnetic cellulose nanocrystal (CNC/Fe_3_O_4_) nanocomposites represent a promising class of materials with potential applications across biomedical, environmental, and energy-related fields due to their tunable magnetic properties, biocompatibility, and functional surface chemistry. Despite this promise, studies elucidating the nature of the binding between CNC and Fe_3_O_4_ are limited, yet such insights are critical for the rational design and optimization of nanocomposites for applications like magnetic hyperthermia, targeted drug delivery, and magnetic separation.

In this work, CNC/Fe_3_O_4_ nanocomposites were synthesized using sulfated CNC (S-CNC) and TEMPO-oxidized CNC (T-CNC) as templates at CNC-to-magnetite mass ratios of 1:2 and 1:4 to systematically investigate how surface chemistry influences magnetite attachment, distribution, and magnetic performance. FTIR confirmed the successful surface oxidation of S-CNC to T-CNC, while XPS analyses revealed interactions between CNC functional groups and Fe_3_O_4_ nanoparticles. In the O 1s spectra, S-CNC/Fe_3_O_4_ nanocomposites exhibited an additional peak around 530.7 eV corresponding to O–Fe bonds, while T-CNC/Fe_3_O_4_ showed a similar O–Fe peak at 530.4 eV. Those peaks are shifted toward higher binding energy compared to bare Fe_3_O_4_ nanoparticles, for which the O–Fe peak appears at 530.1 eV. The shift likely arises from electrostatic interactions between the CNC surface and Fe_3_O_4_, as further supported by DFT, Bader charge, and PDOS analyses. In the Fe 2p spectra, Fe 2p_3_/_2_ peaks in CNC/Fe_3_O_4_ shifted slightly to higher binding energies (711.2–712.0 eV) compared to bare Fe_3_O_4_ (711.0 eV); while the shift is more noticeable for the 1:2 ratio, it suggests electronic interactions with CNC oxygen groups while retaining the Fe^2+^/Fe^3+^ oxidation states and spinel structure. Density functional theory (DFT) provided complementary molecular-level insights, confirming that −OH groups in S-CNC and −COOH groups in T-CNC facilitate adsorption through electrostatic interactions with the Fe_3_O_4_ cluster. The calculations further revealed that CNC surface characteristics influence adsorption energy, with the T-CNC surface (−COOH group site) exhibiting greater susceptibility and stronger interactions than pristine CNC. Bader charge and PDOS analysis revealed that all adsorption is stabilized by electrostatic interaction rather than hydrogen bonding; moreover, ICOHP analysis suggests the strength of the newly formed Fe–O bonds in T-CNC/Fe_3_O_4_ is in the covalent range.

TEM and EDX analyses confirmed Fe_3_O_4_ attachment to the CNC surface, with T-CNC/Fe_3_O_4_ exhibiting a more uniform distribution than S-CNC composites. DLS and zeta potential measurements showed that magnetic CNCs possess improved colloidal stability compared to bare Fe_3_O_4_. VSM measurements revealed superparamagnetic behavior for all nanocomposites, with S-CNC/Fe_3_O_4_ (1:4 and 1:2) displaying higher saturation magnetization than T-CNC/Fe_3_O_4_ counterparts.

Magnetic hyperthermia studies revealed that heating efficiency and specific absorption rate (SAR) increased with both magnetic field strength and Fe_3_O_4_ content. The S-CNC/Fe_3_O_4_ (1:4) and T-CNC/Fe_3_O_4_ (1:4) exhibited higher SAR (W/g-sample) at the highest tested magnetic field (30.4 ± 0.9 kA/m) than their 1:2 counterparts; however when normalized per gram of magnetite, S-CNC/Fe_3_O_4_ (1:2) showed the highest intrinsic SAR, indicating an optimal balance between dispersion and magnetic content, highlighting the positive relationship between shorter Néel and Brownian relaxation times with higher SAR. The T-CNC/Fe_3_O_4_ composites experienced lower magnetization than the S-CNC/Fe_3_O_4_ nanocomposites; however, they achieved comparable heating with superior colloidal stability. These findings highlight the critical role of CNC surface chemistry and nanoparticle loading in optimizing magnetic nanocomposites for hyperthermia and related applications.

Cytotoxicity assays with RAW 264.7 macrophages confirmed that all magnetic CNC nanocomposites showed no toxicity at 20 or 100 μg/mL. At the higher 500 μg/mL concentrations, only the S-CNC/Fe_3_O_4_ (1:4) showed a decrease in MTT reduction activity, but no increase in LDH activity, which instead decreased at this concentration. Overall cellular damages were not observed, yet the specific nanoparticle composition could make a difference in the effects on cellular metabolism. While both assays are general cellular toxicity assays, our results suggest that further investigations are needed to establish the exact cellular responses to the different nanocomposites. Given the favorable safety profile, magnetic CNC nanocomposites are promising candidates for biomedical applications, such as magnetic hyperthermia, targeted drug delivery, and antimicrobial or surface-disinfection platforms, where both magnetic responsiveness and cytocompatibility are essential. This study provides a comprehensive analysis of the structure–property relationships, linking CNC surface chemistry, Fe_3_O_4_ binding, magnetic behavior, and hyperthermia performance, providing valuable insights for the rational design of functional magnetic CNC nanocomposites.

## Supplementary Material



## Notes

STEM images of CNCs and magnetic CNCs; zeta potential and particle size data; TGA-derived magnetite content; bulk properties of CNC and the computed results; adsorption energy, local and total magnetic moment; Bader charge analysis of the atoms involved in the adsorption sites of magnetite and oxygens of the CNC/T-CNC (100) surface; Bader charge analysis of the magnetite cluster atoms (3 Fe and 4 O) before and after the adsorption; Top/side views of CNC and PDOS of CNC/T-CNC surfaces; PDOS and crystal orbital Hamiltonian of the most stable complex; conductometric titration details; magnetite content measurement protocols using TGA data; magnetic parameters of various CNC/Fe_3_O_4_ nanocomposites (PDF)
