# Supplementary material for: Self-Assembly Interactions in Magnetite-Coated Cellulose Nanocrystals: Implications for Magnetic Hyperthermia Applications
Source: ACS Appl Nano Mater. 2026 Apr 7;9(17):7431–47. doi: 10.1021/acsanm.5c05783 (PMC13140134; doi:10.1021/acsanm.5c05783)
Supplement: Supplementary file 1 [file an5c05783_si_001.pdf]

Supporting Information

Self-Assembly Interactions in Magnetite-Coated Cellulose Nanocrystals: Implications for Magnetic Hyperthermia Applications

Mohammad Jahid Hasan,[a] Erin L. McNeill,[a] Kishore Chand,[b] Juganta K. Roy,[c] Monishita Deb,[d] Katherine Schlaak,[d] Sydney Herzog,[e] Yvonne Sun,[e] Sarah Watzman,[d] Esteban E. Ureña-Benavides,[a,] Erick S. Vasquez-Guardado[b,f,*]*

[a] Department of Biomedical Engineering and Chemical Engineering, The University of Texas at San Antonio, 1 UTSA Circle, San Antonio, TX, 78249, USA

[b] Department of Chemical and Materials Engineering, University of Dayton, 300 College Park, Dayton, OH 45469-0256, USA

[c] Department of Chemistry and Physics, West Texas A&M University, Canyon, Texas 79016, USA

[d] Department of Mechanical and Materials Engineering, University of Cincinnati, 2901Woodside Drive, Cincinnati, OH, 45221, USA

[e] Department of Biology, University of Dayton, Dayton, OH 45469, USA

[f] Hanley Sustainability Institute, University of Dayton, 300 College Park, Dayton, OH, 45469, USA

[*] Corresponding authors

Corresponding Authors: Esteban E. Urena-Benavides, Ph.D. and Erick S. Vasquez-Guardado, Ph.D.

Corresponding Authors' emails: esteban.urena-benavides@utsa.edu and evasquez1@udayton.edu

Table of Contents

Supporting Text S1 (Measurement of Magnetite Content in CNC/Fe_3O_4).................................S3
Table S1....S4
Table S2....S4
Table S3....S4
Figure S1. ....S5
Supporting Text S2 (Conductometric Titration of TEMPO-CNC)...S6
Figure S2. ....S7
Figure S3. ....S8
Figure S4. ....S9
Figure S5. ....S10
Figure S6. ....S11
Table S4....S12
Table S5....S13
Table S6....S13
Figure S7. ....S14
Table S7....S15
Figure S8. ....S16

Supporting Text S1 (Measurement of Magnetite Content in CNC/Fe_3O_4)

The magnetic content in the magnetic CNCs (S-CNC/Fe_3O_4 and T-CNC/Fe_3O_4 composites) was calculated using the residual weight of each samples from TGA graph at 600℃ (shown in Figure 3 of the main manuscript). All quantities are given per *initial* unit mass of CNC/Fe_3O_4.

Calculations of magnetite (Fe_3O_4) content:

This derivation assumes the relative amount of weight loss of magnetite or cellulose is not affected by the presence of the other component.

Define Fe_3O_4 content in CNC/Fe_3O_4 = M (gram of Fe_3O_4 / gram CNC/Fe_3O_4)

Therefore, CNC content in magnetic CNCs = 1-M (gram of CNC / gram of CNC/Fe_3O_4)

Now, the Residual weight (RW) of hybrid CNC/Fe_3O_4 NPs with respect to the initial mass is

$$RW_{\mathrm{CNC}/Fe_3O_4} = \left(\frac{mass\ left\ Fe_3O_4}{initial\ mass\ \mathrm{CNC}/Fe_3O_4}\right) + \left(\frac{mass\ left\ CNC}{initial\ mass\ \mathrm{CNC}/Fe_3O_4}\right)$$

$$RW_{\mathrm{CNC}/Fe_3O_4} = \left(\frac{mass\ left\ Fe_3O_4}{initial\ mass\ \ Fe_3O_4}\right)(M) + \left(\frac{mass\ left\ CNC}{initial\ mass\ \ CNC}\right)(1-M)$$

$$RW_{\mathrm{CNC}/Fe_3O_4} = \left(RW_{Fe_3O_4}\right)(M) + (RW_{CNC})(1-M)$$

$$RW_{\mathrm{CNC}/Fe_3O_4} = \left(RW_{Fe_3O_4}\right)(M) + (RW_{CNC}) - (RW_{CNC})(M)$$

$$RW_{\mathrm{CNC}/Fe_3O_4} - RW_{CNC} = (M)\left(RW_{Fe_3O_4} - RW_{CNC}\right)$$

Therefore, $M = \frac{RW_{\mathrm{CNC}/Fe_3O_4} - RW_{CNC}}{RW_{Fe_3O_4} - RW_{CNC}} \times 100\ \%$

Therefore, the magnetite content of CNC/Fe_3O_4 can be calculated from the equations below:

$$M_{SCNC/Fe_3O_4} = Fe_3O_4\ Content\ in\ SCNC/Fe_3O_4 = \frac{RW_{\mathrm{SCNC}/Fe_3O_4} - RW_{SCNC}}{RW_{Fe_3O_4} - RW_{SCNC}} \times 100\ \%$$

$$M_{TCNC/Fe_3O_4} = Fe_3O_4\ Content\ in\ TCNC/Fe_3O_4 = \frac{RW_{\mathrm{TCNC}/Fe_3O_4} - RW_{TCNC}}{RW_{Fe_3O_4} - RW_{TCNC}} \times 100\ \%$$

Where, $RW_{\mathrm{SCNC}/Fe_3O_4}$, RW_{SCNC}, $RW_{Fe_3O_4}$, $RW_{\mathrm{TCNC}/Fe_3O_4}$, and RW_{TCNC} represented the residual weight of S-CNC/Fe_3O_4, bare S-CNC, bare magnetite NPs, T-CNC/Fe_3O_4, and bare T-CNCs respectively at 600˚C. The magnetite content in the CNC/Fe_3O_4 is shown in Table S5.

The residual weight (%) and calculated magnetite (Fe_3O_4) content in the magnetic nanocomposites based on TGA analysis are provided in Table S1.

Table S1. Residual weight (%) and calculated magnetite (Fe_3O_4) content in the magnetic nanocomposites based on TGA analysis.

Sample Name	Residual weight (%)	Magnetite content (%)
S-CNC	15.5 ± 0.5	0
T-CNC	21.9 ± 0.5	0
Bare Fe_3O_4	96.3 ± 0.2	100
S-CNC/Fe_3O_4 1:2	66.8 ± 0.5	63.5 ± 0.7
S-CNC/Fe_3O_4 1:4	86.8 ± 0.5	88.2 ± 0.7
T-CNC/Fe_3O_4 1:2	70.9 ± 0.5	65.8 ± 0.7
S-CNC/Fe_3O_4 1:4	85.4 ± 0.5	85.4 ± 0.7

Table S2. Bulk properties of CNC and the computed results.

	V(Å^3)	a(Å)	b(Å)	c(Å)	α(deg)	β(deg)	γ(deg)
vdW-DF2	655.94	7.57	8.21	10.62	90.0	90.0	96.96
Expt. (295 K)[6]	655.50	7.76	8.20	10.37	90.0	90.0	96.62

Table S3. Surface formation of the three different surfaces of cellulose Iβ in mJ/m^2

Surface of Iβ	$nE_{I\beta}^{Bulk}$, in eV	A, in Å^2	E_{facets}^{slab}, in eV	E_{surf}^{f}, in mJm^{-2}
100	-423.99	87.2	-1059.24	66.9
200		87.2	-1059.23	67.3
110		126.0	-2117.31	167.0

Note 1: Surface formation energy was calculated by using the following equation[4]:

$$E_{surf}^{f} = \frac{E_{facets}^{slab} - nE_{I\beta}^{Bulk}}{2A}$$

where, E_{facets}^{Slab} is the total energy of the perfect surface, $E_{I\beta}^{Bulk}$ is bulk energy per formula unit in the bulk, n is the number of formula unit in supercell and E_{Surf}^{f} is surface formation energy.

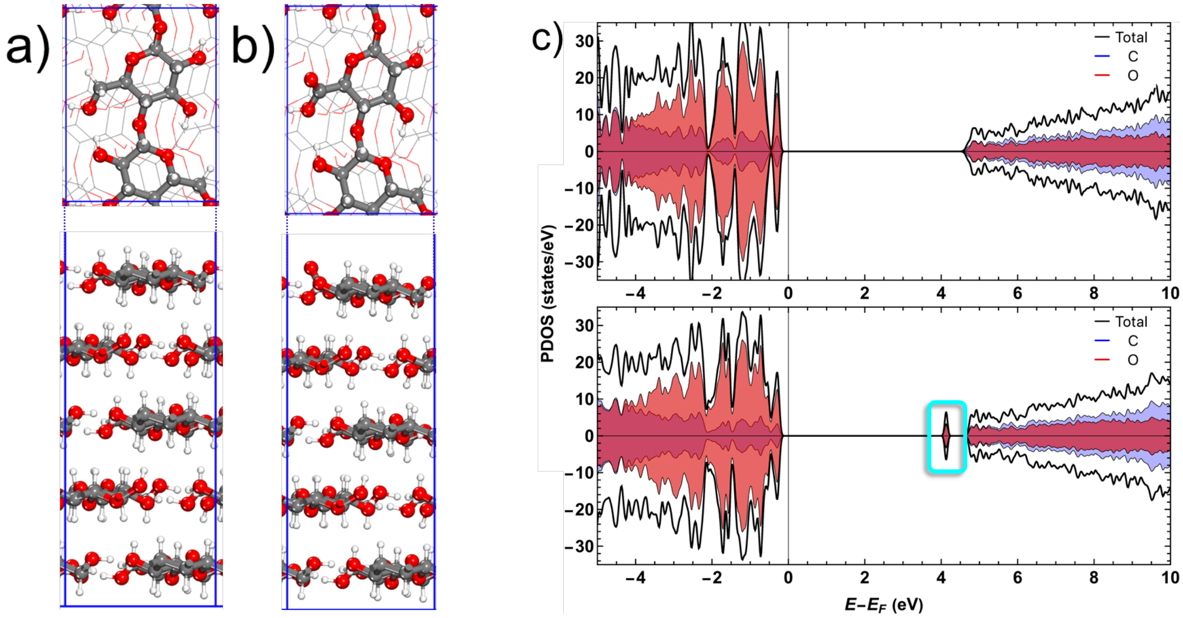

Figure S1. Top and side view of the (a) S-CNC (100) and (b) T-CNC (100). System is consisting of five layers of Iß-cellulose and the bottommost three layers were fixed. c) The PDOS of the S-CNC and T-CNC surfaces. The cyan box indicates the peak of tempo-oxidized -COOH group.

Supporting Text S2 (Conductometric Titration of TEMPO-CNC)

A 40 mL, 1 wt% TEMPO-CNC dispersion was first acidified with 0.1 M HCl. Excess acid was removed by dialysis using a suitable membrane, replacing the water daily for three days until the dialysis water reached a constant pH, indicating complete removal of residual acid. The concentration of the dialyzed TEMPO-CNC was then determined, and a 160 mL, 0.1 wt% TEMPO-CNC suspension in 1 mM NaCl was prepared accordingly, and sonicated using a Q700 probe sonicator from Qsonica LLC (Newtown, CT) (amplitude 30; 20 s ON and 20 s OFF).

For conductometric titration, 0.01 M NaOH was added to the TEMPO-CNC suspension in 0.5 mL increments at 2 min intervals, with the electrical conductivity measured after each addition using a VWR Symphony B30PCI benchtop pH and conductivity meter (VWR, Radnor, PA, USA). Three distinct trends were observed: (i) an initial decrease in conductivity corresponding to neutralization of sulfonate (-HSO_3) groups on the CNC surface, (ii) a plateau associated with neutralization of the weaker carboxyl (-COOH) groups, and (iii) a subsequent increase due to excess base. The titration was stopped when the increasing trend length approximately matched the initial decreasing trend.

The volume of NaOH corresponding to the neutralization of -HSO_3 and –COOH groups based on the equations below:

$$\%S = \left(\frac{V_1 \times C_{NaOH} \times MW_{sulphur}}{m_{susp} \times C_{susp}}\right) \times 100 \text{...(Eqn. S1)}$$

Where, V_1 is the volume of base required to neutralize the -HSO_3,

C_{NaOH} is the concentration of base used for titration,

$MW_{Sulphur}$ is the molecular weight of Sulphur,

m_{susp}, and C_{susp} are mass and concentration (mass ratio of CNC and the suspension) of CNC suspension used for titration respectively.

$$\%COOH = \left(\frac{(V_2 - V_1) \times C_{NaOH} \times MW_{COOH}}{m_{susp} \times C_{susp}}\right) \times 100 \text{ ....(Eqn. S2)}$$

Where, $(V_2 - V_1)$ is the volume of base required to neutralize -COOH group,

and, MW_{COOH} is the molecular weight of COOH.

The Figure S4 below shows the titration graph.

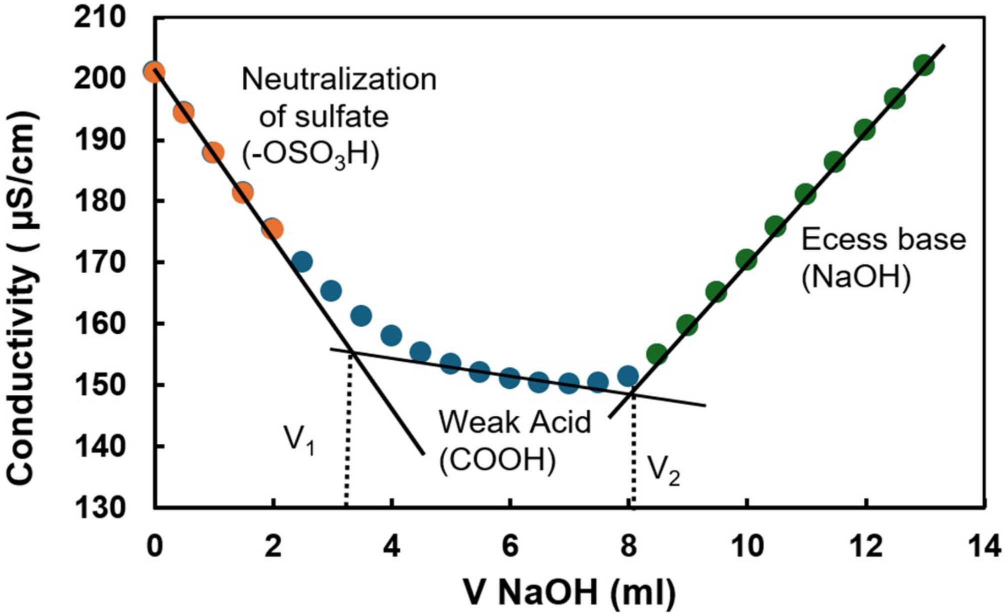

Figure S2. Conductometric titration of Tempo-mediated oxidized CNC (T-CNC).

Results: Based on conductometric titration and using the Eqn. S1 and S2, the results are summarized below:

Sulfur content: 0.64 wt%

OSO_3H content: 200 mmol OSO_3H per kg CNC

COOH content: 1.379 wt%

COOH Content: 306.25 mmol COOH per kg CNC

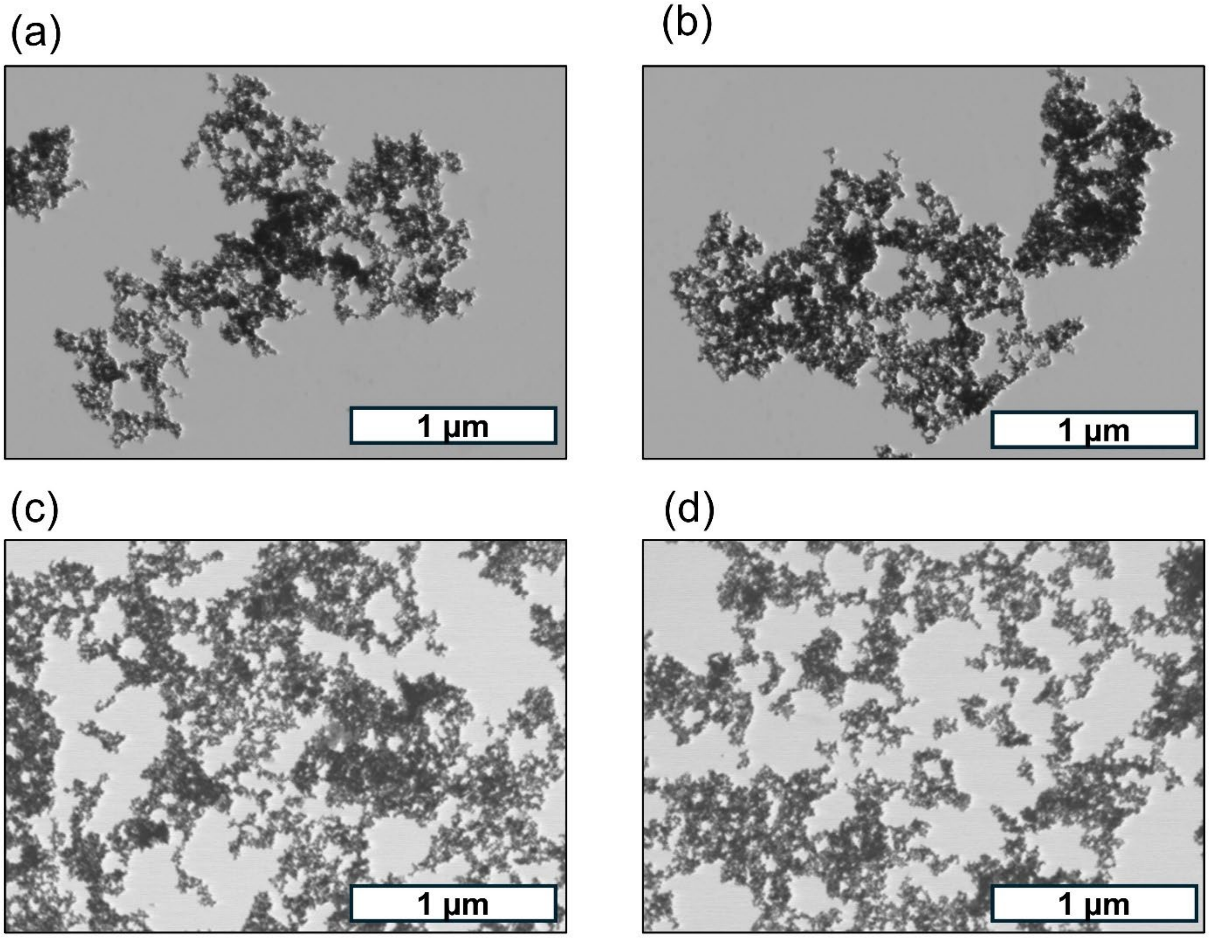

Figure S3. Bright-field STEM images of physically mixed samples of Fe_3O_4 and CNCs after magnetic washing for ratios (a) S-CNC/Fe_3O_4 1:2 mixture, (b) S-CNC/Fe_3O_4 1:4 mixture, (c) T-CNC/Fe_3O_4 1:2 mixture, and (d) T-CNC/Fe_3O_4 1:4. No CNCs are observed in the images, indicating a lack of attachment between CNC and Fe_3O_4 during simple physical mixing. These results suggest that in-situ co-precipitation of CNC/Fe_3O_4 is required to achieve strong attachment.

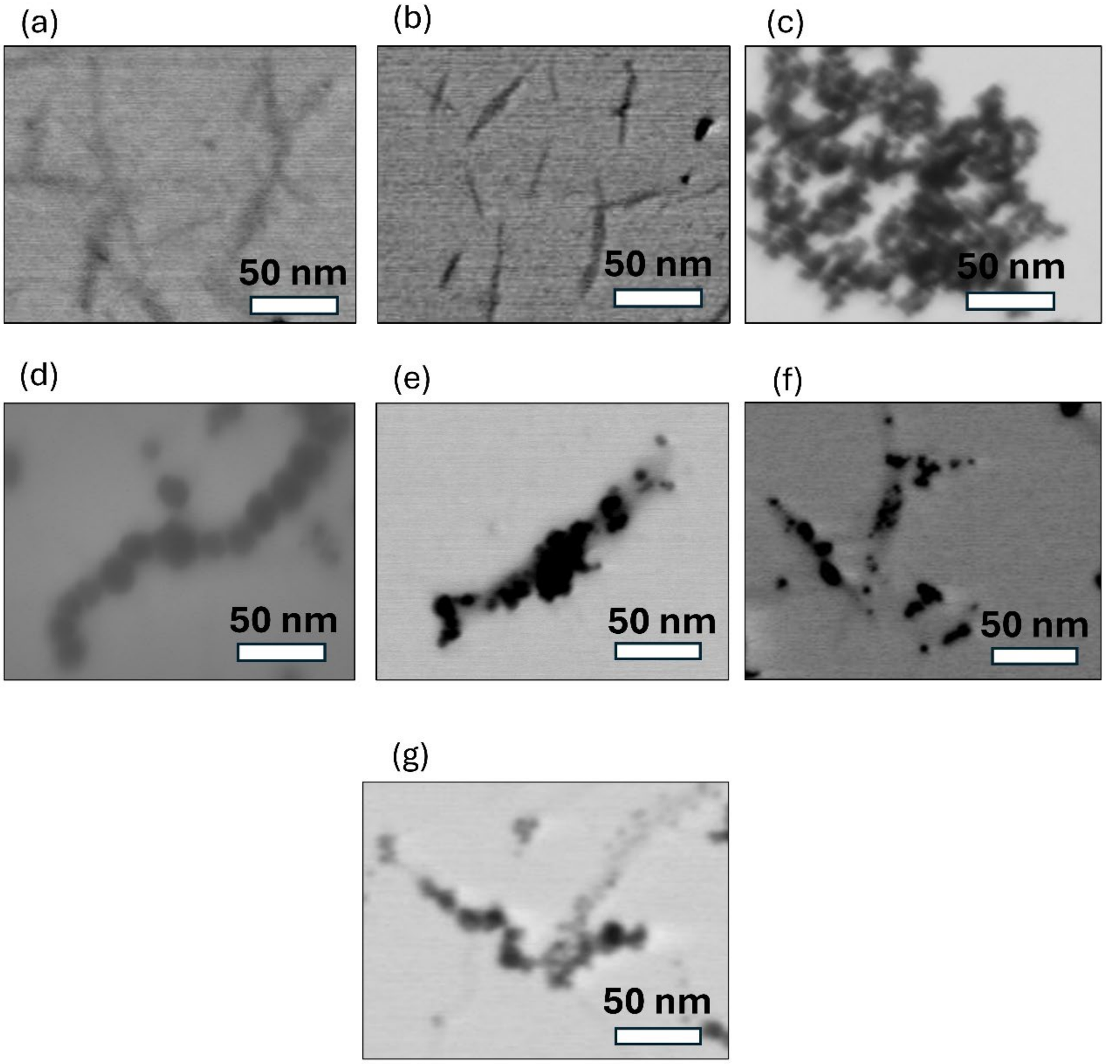

Figure S4. High magnification bright-field STEM images of (a) S-CNC; (b) T-CNC; (c) bare Fe_3O_4 nanoparticles; (d) S-CNC/Fe_3O_4 1:2; (e) S-CNC/Fe_3O_4 1:4; (f) T-CNC/Fe_3O_4 1:2; (g) T-CNC/$Fe_3O_4$1:4. Scale bars in all panels = 2 μm.

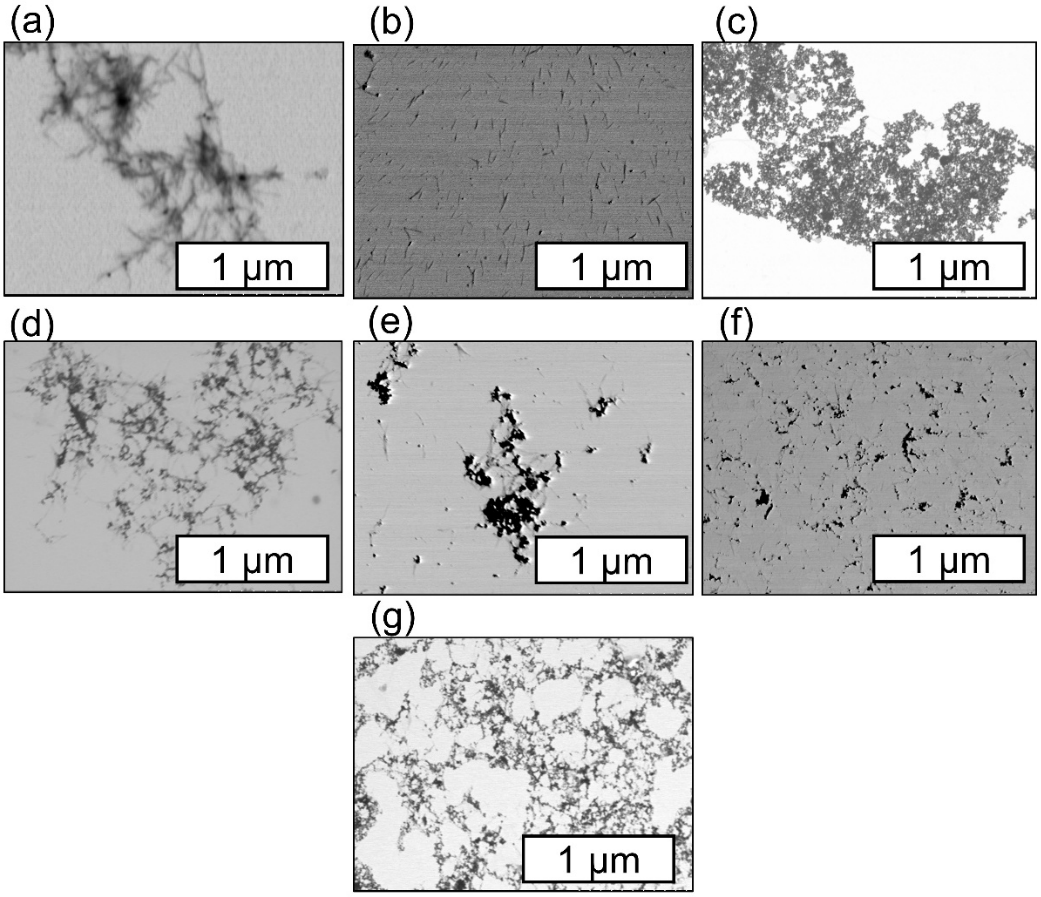

Figure S5. Bright-field STEM images of (a) S-CNC; (b) T-CNC; (c) bare Fe_3O_4 nanoparticles; (d) S-CNC/Fe_3O_4 1:2; (e) S-CNC/Fe_3O_4 1:4; (f) T-CNC/Fe_3O_4 1:2; (g) T-CNC/$Fe_3O_4$1:4. Scale bars in all panels = 2 μm.

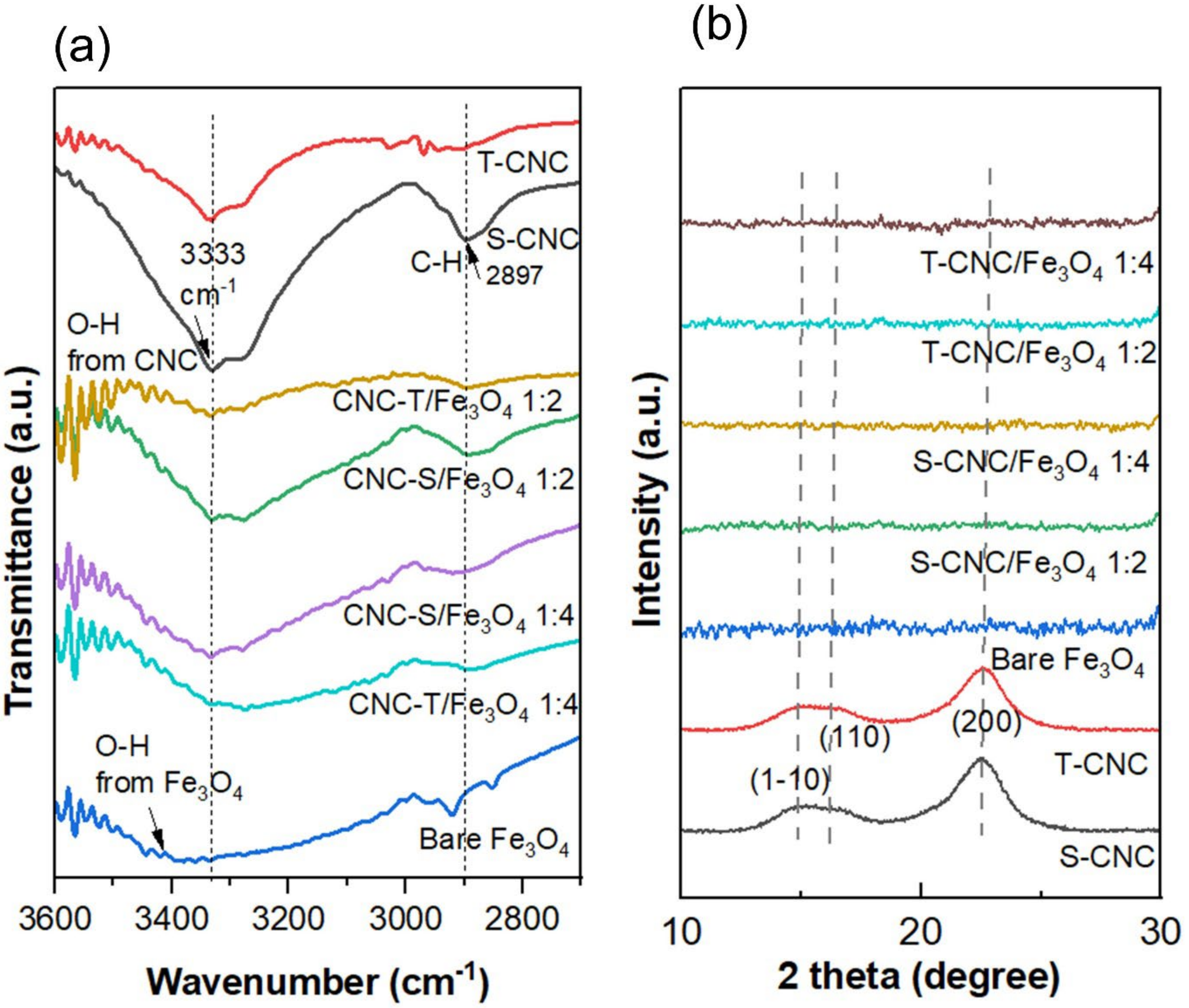

Figure S6. (a) FT-IR spectroscopy of CNCs, bare Fe_3O_4 and CNC/Fe_3O_4 nanocomposites at 2700 to 3600 cm^{-1}, showing OH peak from bare Fe_3O_4 and CNCs. (b) XRD of CNCs, bare Fe_3O_4 and CNC/Fe_3O_4 nanocomposites.

Table S4. Computed adsorption energy in eV per (Fe_3O_4) formula unit, local and total magnetic moment in Bohr magnetons (μ_B) of the $(Fe_3O_4)_{n=1}$ on S-CNC (100) and T-CNC (100).

adsorption sites	$E_{ads}/(Fe_3O_4)$, eV		S-CNC, μ_B			T-CNC, μ_B		
	S-CNC	T-CNC	Fe (1/2/3)	O_{sites}	Total	Fe (1/2/3)	O_{sites}	Total
C6	-0.39	-	-	-	5.51	-	-	-
O2	-0.99	-0.82	+3.13<br>+1.05<br>+1.01	O2 = 0.04	5.58	+3.02<br>-1.13<br>+2.97	O2 = 0.02	5.46
O5	-1.54	-	+0.70<br>+3.16<br>+2.66	O5 = 0.03	7.36	-	-	-
Ot	-	-1.93				-3.03<br>+3.06<br>+3.02	Ot = −0.06	3.57
O6/O1	-1.88	-	+3.21<br>+3.20<br>+0.39	O6 = 0.05<br>O1 = 0.03	7.49	-	-	-
O5/Ot	-	-2.03	-	-	-	-3.18<br>+3.02<br>+3.18	O5 = −0.03<br>Ot = 0.02	3.58
O1/O5	-1.11	-	+3.51<br>+3.18<br>+0.20	O1 = 0.02<br>O5 = 0.03	7.43	-	-	-
O1/O6	-	-1.33	-	-	-	+3.55<br>+3.18<br>+0.18	O1 = 0.02<br>O6 = 0.04	7.44

Table S5. Bader charge analysis of the atoms involved in the adsorption sites of magnetite and oxygens of the CNC/t-CNC (100) surface.

system	Bader charge $\lvert e^- \rvert$					
	**Fe**	**O1**	**O2**	**O6**	**Ot**	**O5**
CNC (100)		-0.884	-0.839	-0.823		-0.789
$t-CNC$ (100)		-0.858	-0.733	-0.728	-0.979	-0.735
$CNC/O2-Fe$	1.141		-0.884			
$CNC/O5-Fe$	0.712					-0.708
$CNC/O6/O1-2Fe$	1.081<br>1.027	-0.848		-0.884		
$CNC/O1/O5-Fe$	1.197	-0.891				-0.755
$t-CNC/Ot-Fe$	1.241				-1.002	
$t-CNC/O2-Fe$	1.156		-0.751			
$t-CNC/O1-Fe$	1.147					
$t-CNC/O5/Ot-Fe$	0.877				-0.902	-0.719

Table S6. Bader charge analysis of the magnetite cluster atoms (3 Fe and 4 O) before and after the adsorption. Fe_{tot}, O_{tot} and FeO_{net} indicates the total positive charge of 3 Fe atoms, total negative charge of 4 O atoms, and the net charge ($Fe_{tot} + O_{tot}$) of the cluster, respectively.

system	Bader charge $\lvert e^- \rvert$									
	**Fe_1**	**Fe_2**	**Fe_3**	$\mathbf{Fe_{tot}}$	**O_1**	**O_2**	**O_3**	**O_4**	$\mathbf{O_{tot}}$	$\mathbf{FeO_{net}}$
Magnetite (Fe_3O_4)	1.078	1.312	1.095	3.485	-0.857	-0.769	-1.006	-0.854	-3.486	-0.001
**after the adsorption**										
$S-CNC/C6-Fe$	0.528	1.075	1.393	2.996	-0.625	-0.630	-0.917	-0.644	-2.816	0.180
$S-CNC/O2-Fe$	1.220	1.141	0.981	3.342	-0.794	-0.820	-0.869	-0.765	-3.248	0.093
$S-CNC/O5-Fe$	0.712	1.336	1.282	3.330	-0.902	-0.869	-0.793	-0.765	-3.329	0.001
$S-CNC/O6/O1-2Fe$	1.081	1.027	1.190	3.298	-0.901	-0.818	-0.663	-0.814	-3.197	0.101
$S-CNC/O1/O5-Fe$	1.197	1.415	0.873	3.485	-0.871	-0.863	-0.818	-0.918	-3.470	0.015
$T-CNC/Ot-Fe$	1.241	1.358	1.293	3.892	-0.889	-0.979	-0.913	-0.992	-3.773	0.119
$T-CNC/O2-Fe$	1.156	1.172	0.760	3.088	-0.717	-0.635	-0.882	-0.725	-2.959	0.130
$T-CNC/O1-Fe$	1.147	1.346	0.966	3.459	-0.917	-0.807	-0.856	-0.884	-3.464	-0.005
$T-CNC/O5/Ot-Fe$	0.877	1.198	0.875	2.950	-0.802	-0.808	-0.675	-0.654	-2.940	0.010

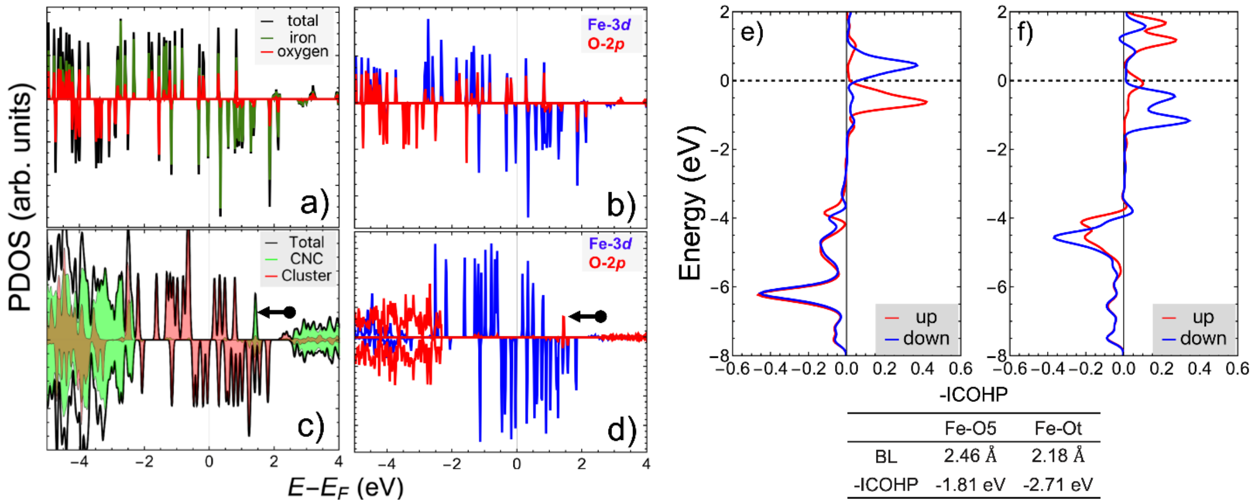

	Fe-O5	Fe-Ot
BL	2.46 Å	2.18 Å
-ICOHP	-1.81 eV	-2.71 eV

Figure S7. DFT computed PDOS of the pure Fe_3O_4 cluster (a, b) and PDOS of the most stable complex of T-CNC/Fe_3O_4 (c, d). Crystal orbital Hamiltonian population of the stable composite (e,f). The O 2p orbital in (d) is only for the CNC. The Fermi level is indicated by gray line and the peak indicated by the black arrow represents the oxygen from the TEMPO-oxidized carboxylic group.

Table S7. Magnetic parameters of various CNC/Fe_3O_4 nanocomposites.

Sample name	Mr (emu/g-sample)	Mr (emu/g-Fe_3O_4)	Hc (Oe)	Ms (emu/g-sample)	Ms (emu/g-Fe_3O_4)	T_B (K)
S-CNC/Fe_3O_4 1:2	2.5 ± 0.2	3.9 ± 0.3	24.3 ± 2.7	48.9 ± 0.3	77.0 ± 1.0	112.1 ± 0.1
S-CNC/Fe_3O_4 1:4	1.9 ± 0.2	2.1 ± 0.2	17.9 ± 3.1	68.5 ± 0.4	77.6 ± 0.8	112.1 ± 0.1
T-CNC/Fe_3O_4 1:2	1.8 ± 0.2	2.8 ± 0.3	33.6 ± 2.8	43.8 ± 0.4	66.5 ± 0.9	99.5 ± 0.1
T-CNC/Fe_3O_4 1:4	1.3 ± 0.2	1.5 ± 0.2	16.8 ± 2.9	51.4 ± 0.3	60.1 ± 0.6	112.1 ± 0.1
Bare Fe_3O_4	1.2 ± 0.3	1.2 ± 0.3	14.3 ± 4.3	82.5 ± 0.5	82.5 ± 0.5	112.1 ± 0.1

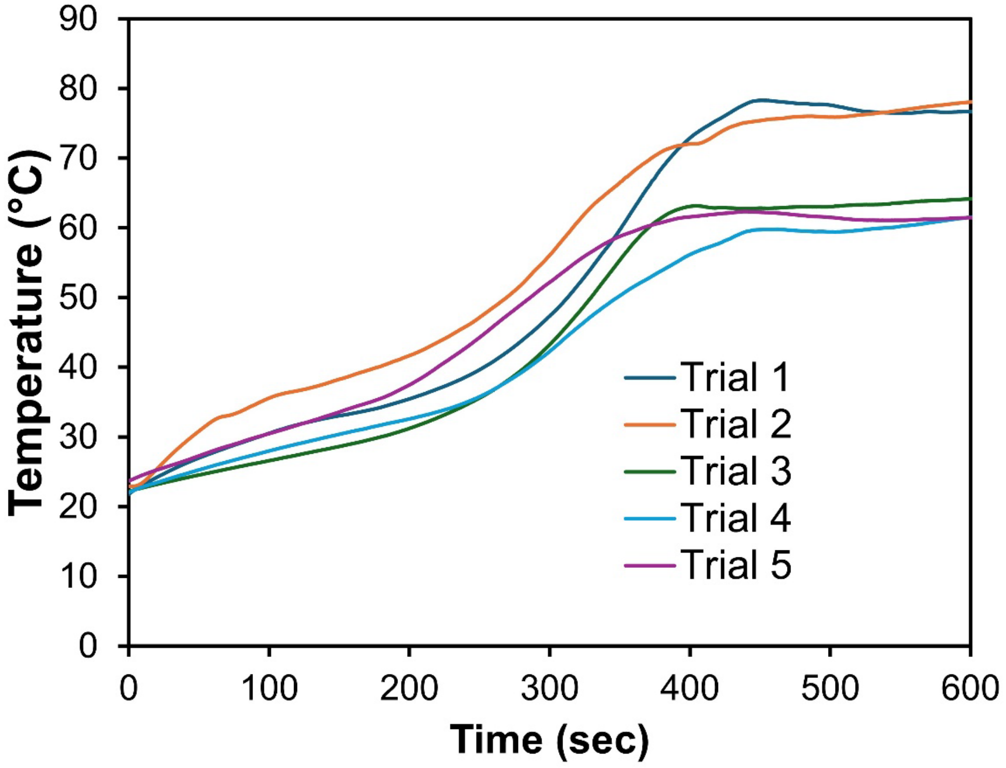

Figure S8. Magnetic hyperthermia heating profiles of bare magnetite nanoparticle (3 wt% concentration in water) under a 155 ± 4 kHz alternating magnetic field with amplitudes of 30.4 ± 0.9 kA/m.
